# Single-cell transcriptomics reveals correct developmental dynamics and high-quality midbrain cell types by improved hESC differentiation

**DOI:** 10.1016/j.stemcr.2022.10.016

**Published:** 2022-11-17

**Authors:** Kaneyasu Nishimura, Shanzheng Yang, Ka Wai Lee, Emilía Sif Ásgrímsdóttir, Kasra Nikouei, Wojciech Paslawski, Sabine Gnodde, Guochang Lyu, Lijuan Hu, Carmen Saltó, Per Svenningsson, Jens Hjerling-Leffler, Sten Linnarsson, Ernest Arenas

**Affiliations:** 1 Department of Medical Biochemistry and Biophysics, Karolinska Institutet, 171 77 Stockholm, Sweden; 2 Department of Clinical Neuroscience, Karolinska University Hospital, 171 77 Stockholm, Sweden

**Keywords:** human, midbrain, development, embryonic stem cell, dopaminergic neuron, function, single-cell RNA sequencing, Parkinson's disease

## Abstract

Stem cell technologies provide new opportunities for modeling cells in health and disease and for regenerative medicine. In both cases, developmental knowledge and defining the molecular properties and quality of the cell types is essential. In this study, we identify developmental factors important for the differentiation of human embryonic stem cells (hESCs) into functional midbrain dopaminergic (mDA) neurons. We found that laminin-511, and dual canonical and non-canonical WNT activation followed by GSK3β inhibition plus FGF8b, improved midbrain patterning. In addition, neurogenesis and differentiation were enhanced by activation of liver X receptors and inhibition of fibroblast growth factor signaling. Moreover, single-cell RNA-sequencing analysis revealed a developmental dynamics similar to that of the endogenous human ventral midbrain and the emergence of high-quality molecularly defined midbrain cell types, including mDA neurons. Our study identifies novel factors important for human midbrain development and opens the door for a future application of molecularly defined hESC-derived cell types in Parkinson disease.

## Highlights

• A human midbrain-based protocol improves the generation of functional hESC-mDA neurons
• Laminin 511, dual WNT5A-CHIR, and FGF8b enhance midbrain patterning of hESCs
• LXR activation and FGF inhibition improve midbrain neurogenesis and differentiation
• scRNA-seq revealed high-quality hESC-derived cell types, resembling the human midbrain

## Graphical abstract

**Figure:**
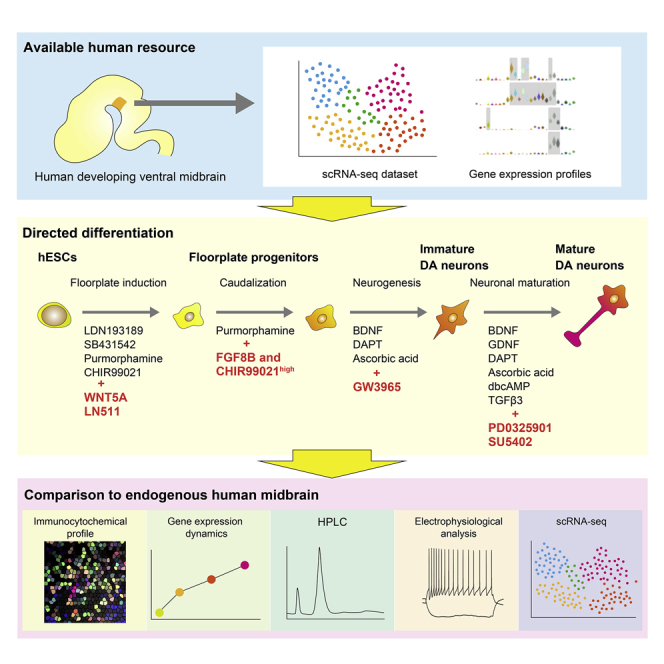

## Teaser

In this article, Arenas and colleagues present a new hESC differentiation protocol to generate functional mDA neurons that recapitulates key features of human ventral midbrain development. The function of developmental factors is examined, and the quality of hESC-derived cell types is compared with human midbrain standards by scRNA-seq. Implications for future PD cell replacement therapy are discussed.

## Introduction

Midbrain dopaminergic (mDA) neurons are known to control several important functions in humans, such as voluntary movement, cognition, motivation, and reward. Among them, mDA neurons of the substantia nigra pars compacta (SNc) project to the caudate-putamen and form the nigrostriatal pathway, which controls voluntary movements. The loss of SNc DA neurons and of dopamine in the caudate-putamen is a defining feature of Parkinson disease (PD) (Damier et al., 1999), a neurodegenerative disorder characterized by paucity of movements, tremor, rigidity, and loss of postural control (Lees et al., 2009). However, the cause of PD is largely unknown, and current treatments are symptomatic and lose efficiency with time.

Progress in understanding the molecular logic and mechanisms controlling mDA neuron development has led to important developments in different areas of stem cell biology, including PD modeling, drug screening, and personalized therapeutics (Caiazza et al., 2020), as well as PD cell replacement therapy (Adler et al., 2019; Arenas et al., 2015; Doi et al., 2020; Kikuchi et al., 2017; Kim et al., 2021; Kirkeby et al., 2017; Moriarty et al., 2022; Schweitzer et al., 2020; Tao et al., 2021). mDA neurons are currently thought to derive from radial glia-like progenitor cells at the caudal and ventral end of the midbrain floor plate (Bonilla et al., 2008; Ono et al., 2007). This area is controlled by signals derived from two organizing centers, the midbrain-hindbrain boundary (MHB) and the floor plate (Wurst et al., 2001). One of the most critical signaling events in mDA neuron development is the activation of the Wnt/β-catenin pathway by Wnt1, a morphogen derived from these two centers (Arenas, 2014). Wnt1 controls several aspects of mDA neuron development, such as anterior-posterior patterning (McMahon and Bradley, 1990; Thomas and Capecchi, 1990), the specification of mDA progenitors (Prakash et al., 2006), and the induction of mDA neurogenesis in the midbrain floor plate (Andersson et al., 2013). Accordingly, activation of this pathway in human pluripotent stem cells (hPSCs) by glycogen synthase kinase (GSK)3β inhibitors, such as CHIR99021, has led to significant improvements in protocols for the generation of mDA neurons (Denham et al., 2012; Doi et al., 2014; Kim et al., 2021; Kirkeby et al., 2012; Kriks et al., 2011). However, there are multiple additional developmental factors and signaling pathways known to control mDA neuron development in mice, whose function in human midbrain development remains to be examined. One of them is Wnt5a, a morphogen known to promote midbrain morphogenesis, neurogenesis, and mDA progenitor differentiation in the developing mouse midbrain (Andersson et al., 2008; Castelo-Branco et al., 2003). Wnt5a is known to activate the Wnt/planar cell polarity/Rac1 (Wnt/PCP/Rac1) pathway in mDA progenitors and neurons (Andersson et al., 2008; Čajánek et al., 2013; Parish et al., 2008). Moreover, analysis of double *Wnt1* and *Wnt5a* knockout mice revealed a complex interplay between these two pathways, which controls diverse aspects of ventral midbrain development (Andersson et al., 2013; Arenas, 2014). Another interesting pathway that remains to be implemented in advanced protocols for mDA differentiation of human embryonic stem cells (hESCs) is activation of the nuclear receptors NR1H3 and NR1H2 (also known as liver X receptor α and β, LXRs), which control not only lipid metabolism but also mDA neurogenesis both *in vitro* and *in vivo* (Sacchetti et al., 2009; Theofilopoulos et al., 2013; Toledo et al., 2020). Finally, one additional component that we examined is the midbrain-specific extracellular matrix protein, laminin 511 (LN511), which is known to expand hPSC-derived mDA progenitors (Doi et al., 2014; Kirkeby et al., 2017) and differentiate neuroepithelial stem cells (Zhang et al., 2017), but it is unclear whether full-length LN511 can control progenitor identity and mDA differentiation in hESCs.

Single-cell RNA-sequencing (scRNA-seq) has provided very powerful and unbiased insights into the cell types and gene expression profiles in the developing human ventral midbrain (Birtele et al., 2020; La Manno et al., 2016). In our study, we used scRNA-seq data (La Manno et al., 2016) as a blueprint of the developmental dynamics and the cell types physiologically found in the developing midbrain *in vivo*, as well as a reference dataset to evaluate cell composition and quality of cell types generated by hESCs during mDA differentiation. Four different types of endogenous human progenitors have been found in the endogenous human midbrain floor plate: the ventral midline progenitor (ProgM), medial floor plate progenitor (ProgFPM), lateral floor plate progenitor (ProgFPL), and neuronal progenitor (NProg). In addition, two radial glia-like cells (Rgl1 and Rgl3) were found enriched in the ventral midbrain floor plate. Moreover, four of these cell types (ProgM, ProgFPM, ProgFPL, and Rgl1) are known to selectively express key factors such as the morphogen *WNT1,* and the transcription factors *LMX1A*, *OTX2,* and *FOXA2,* all of which are required for the specification of mDA progenitors and for mDA neuron development (Andersson et al., 2006; Ferri et al., 2007; Puelles et al., 2004). Other cells of interest are the neuronal progenitor (NProg) and the first postmitotic cell of the mDA lineage, the medial neuroblast (NbM), both of which express genes either involved in or required for mDA neurogenesis, such as *NEUROD1* and *NEUROG2* (also known as *NGN2*) (Kele et al., 2006), respectively. The NbM also expresses the nuclear receptor *NR4A2* (*NURR1*), which is required for mDA neuron development (Zetterström et al., 1997). *NR4A2* is also expressed in the three embryonic mDA neuron subpopulations (DA0, DA1, and DA2) (La Manno et al., 2016), together with tyrosine hydroxylase (*TH*) and transcription factors required for mDA development, such as Engrailed 1 (*EN1*) (Simon et al., 2001), Pre-B-cell leukemia homeobox 1 (*PBX1*) (Villaescusa et al., 2016), and Pituitary homeobox 3 (*PITX3*) (Nunes et al., 2003). However, despite all this knowledge, the precise cell composition and quality of hESC-derived midbrain cell types remains to be compared with endogenous single-cell standards and is largely undefined.

In this study, we leverage existing human scRNA-seq data and functional analysis of the developing mouse midbrain to explore whether three key developmental components (WNT5A, LXR, and LN511) can improve the generation of mDA neurons from hESCs. We carefully monitor pathway activation by synchronizing gene expression in hESCs differentiating into mDA neurons with that in endogenous human ventral midbrain development. We found that dual activation of Wnt/β-catenin and Wnt/PCP/Rac1 with CHIR99021 and WNT5A, respectively, together with activation of LXRs with a synthetic ligand and extended use of LN511, improves mDA differentiation of hESCs. Moreover, scRNA-seq allowed us to define the quality of the hESC-derived cells compared with the endogenous human ventral midbrain standards. We found that our human development-based protocol recapitulates key features of human midbrain development, including the generation of functional mDA neurons and cell types similar to those in the developing human ventral midbrain. Our study thus defines the function of developmental factors during human midbrain differentiation of hESCs and shows their implementation improves the composition and quality of hESC-derived mDA cultures.

## Results

### Efficient induction of midbrain floor plate progenitors by LN511 and dual WNT activation

**Figure 1:**
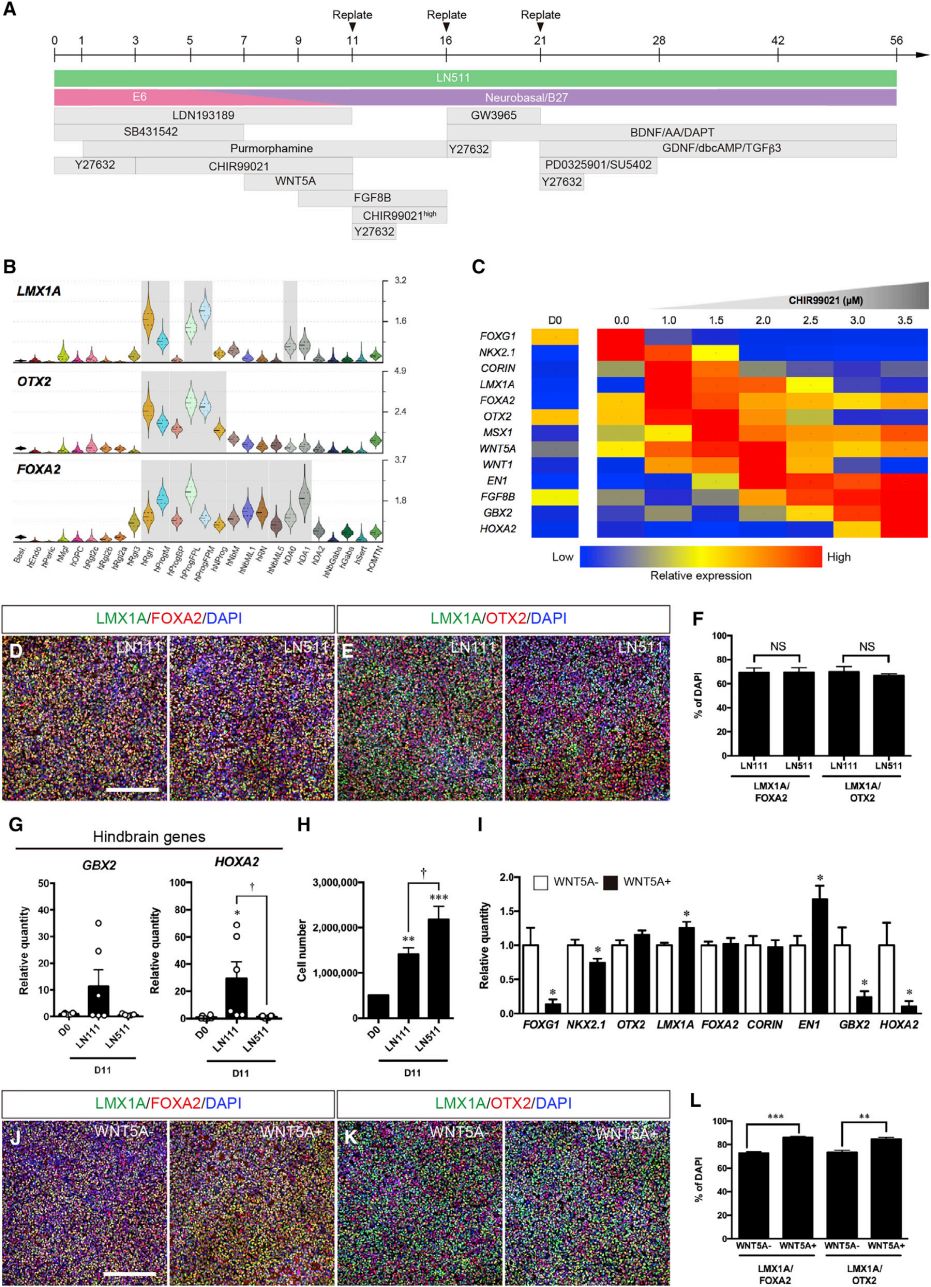
Induction of floor plate progenitors from hESCs at day 11 using a new developmental-based protocol (A) Schematic of the differentiation protocol for mDA neurons. (B) Violin plots of *LMX1A*, *FOXA2*, and *OTX2* generated from scRNA-seq data of developing human ventral midbrain shown across corresponding cell types. Right axis shows absolute molecular counts. Gray, enriched over baseline with posterior probability >99.8%. For cell type nomenclature, see La Manno et al. (2016). (C) Gene expression profile of differentiated cells according to CHIR99021 concentration at day 11. (D and E) Immunostaining of LMX1A^+^;FOXA2^+^ cells (D) and LMX1A^+^;OTX2^+^ cells (E) at day 11. Scale bar, 200 μm. (F) Quantification of LMX1A^+^;FOXA2^+^ cells and LMX1A^+^;OTX2^+^ cells. NS, not significant (n = 4 independent experiments). (G) qPCR analysis of *GBX2* and *HOXA2* in cells differentiating on either LN111 or LN511. ^∗^p < 0.05 versus D0; †p < 0.05 versus LN511 (n = 6 independent experiments). (H) Cell yield by LN111 or LN511 at day 11. ^∗∗^p < 0.01, ^∗∗∗^p < 0.001 versus D0; †p < 0.05 versus LN511 (n = 6 independent experiments). (I) qPCR analysis of differentiated cells in the presence/absence of WNT5A at day11 (n = 5–9 independent experiments). ^∗^p < 0.05 versus WNT5A(−) condition. (J and K) Immunostaining of LMX1A^+^; FOXA2^+^ cells (J) and LMX1A^+^;OTX2^+^ cells (K) at day 11. Scale bar, 200 μm. (L) Quantification of LMX1A^+^;FOXA2^+^ cells and LMX1A^+^;OTX2^+^ cells. ^∗∗^p < 0.01, ^∗∗∗^p < 0.001 versus WNT5A(−) condition (n = 4 independent experiments).

Human ESCs were cultivated in chemically defined medium with the dual Smad inhibitors LDN193189 and SB431542 to promote neural induction (Chambers et al., 2009), the Shh agonist purmorphamine to ventralize (Kriks et al., 2011), and the GSK3β inhibitor CHIR99021 to activate Wnt/βcatenin signaling and achieve caudal midbrain identity (Figure 1A) (Kim et al., 2021; Kirkeby et al., 2012; Kriks et al., 2011). ScRNA-seq data of the developing human ventral midbrain was used to monitor both midbrain patterning and the emergence of midbrain progenitor markers (*LMX1A, FOXA2,* and *OTX2*), which are expressed by four different cell types, Rgl1, ProgM, ProgFPL, and ProgFPM (Figure 1B) (La Manno et al., 2016). qPCR analysis at day 11 (Figures 1C and S1A) showed that 2.0–3.5 μM CHIR99021 induced the expression of hindbrain marker genes (*FGF8B*, *GBX2,* and *HOXA2*), while ventral midbrain genes (*LMX1A, FOXA2, OTX2,* and *CORIN*) were upregulated by 1.0–1.5 μM CHIR99021. Moreover, 1.5–2.0 μM CHIR99021 increased the expression of caudal-ventral midbrain genes (*MSX1, WNT5A, WNT1,* and *EN1*). Since mDA progenitors are enriched in the caudal midbrain (Kirkeby et al., 2017), we chose 1.5 μM CHIR99021 as a baseline to examine the possible function of factors whose function in early mDA progenitor patterning has not yet been fully established, such as LN511 and WNT5A.

We first focused on the extracellular matrix protein LN511, which is enriched in the developing human ventral midbrain and is known to promote the differentiation and survival of mDA neurons (Zhang et al., 2017). hESCs were cultivated on good manufacturing practice (GMP)-grade LN111 or LN511, until day 11. In both cases ≈70% of the cells were LMX1A^+^;FOXA2^+^/DAPI^+^ cells and LMX1A^+^;OTX2^+^/DAPI^+^ (Figures 1D–1F) and the pluripotent stem cell markers *NANOG* and *POU5F1* drastically decreased (Figure S1B). However, LN511 but not LN111 decreased the expression of hindbrain markers such as *GBX2* and *HOXA2* (Figure 1G). In addition, a greater yield of mDA progenitor cells was obtained with LN511 than on LN111 (Figure 1H). Thus, our results show that LN511 efficiently expands midbrain progenitors and prevents the expression of hindbrain patterning genes.

We next investigated the function of WNT5A, a morphogen co-expressed with WNT1 in the four candidate human ventral midbrain DA progenitors (Rgl1, ProgM, ProgFPM, and ProgFPL) (La Manno et al., 2016). Since Wnt1 and Wnt5a are known to cooperate to promote mDA neuron development *in vitro* and *in vivo* (Andersson et al., 2013; Castelo-Branco et al., 2003), we performed a dual WNT activation of hESCs with CHIR99021 and WNT5A from day 7 to day 11 and then examined patterning markers. qPCR analysis at day 11 revealed a significant decrease in the expression of *FOXG1*, *NKX2.1*, *GBX2,* and *HOXA2* and a significant increase in *LMX1A* and *EN1* after treatment with WNT5A (100 ng/mL) (Figure 1I). Moreover, WNT5A also increased the proportion of LMX1A^+^;FOXA2^+^/DAPI^+^ and LMX1A^+^;OTX2^+^/DAPI^+^ cells in a significant manner, from 72.7% ± 1.2% to 86.1% ± 0.7% and from 73.5% ± 1.7% to 84.6% ± 1.7%, respectively (Figures 1J–1L). This regulation was specific, as it did not change LMX1A^+^;CORIN^+^ immunostainig or the proportion of CORIN^+^/DAPI^+^ cells (Figures S1C and S1D), two markers expressed in ProgM (La Manno et al., 2016). These results indicate that WNT5A, in combination with CHIR99021 (1.5 μM), improves the induction of mDA progenitors compared with CHIR99021 alone at the expense of more anterior and posterior fates. Moreover, comparable midbrain patterning was confirmed in three different hESC lines, HS401, HS975, and HS980 (Figures S1E–S1H), indicating that the effects of dual WNT activation are both specific and robust.

### Specification of caudal-ventral midbrain domain by CHIR99021 and fibroblast growth factor 8b

**Figure 2:**
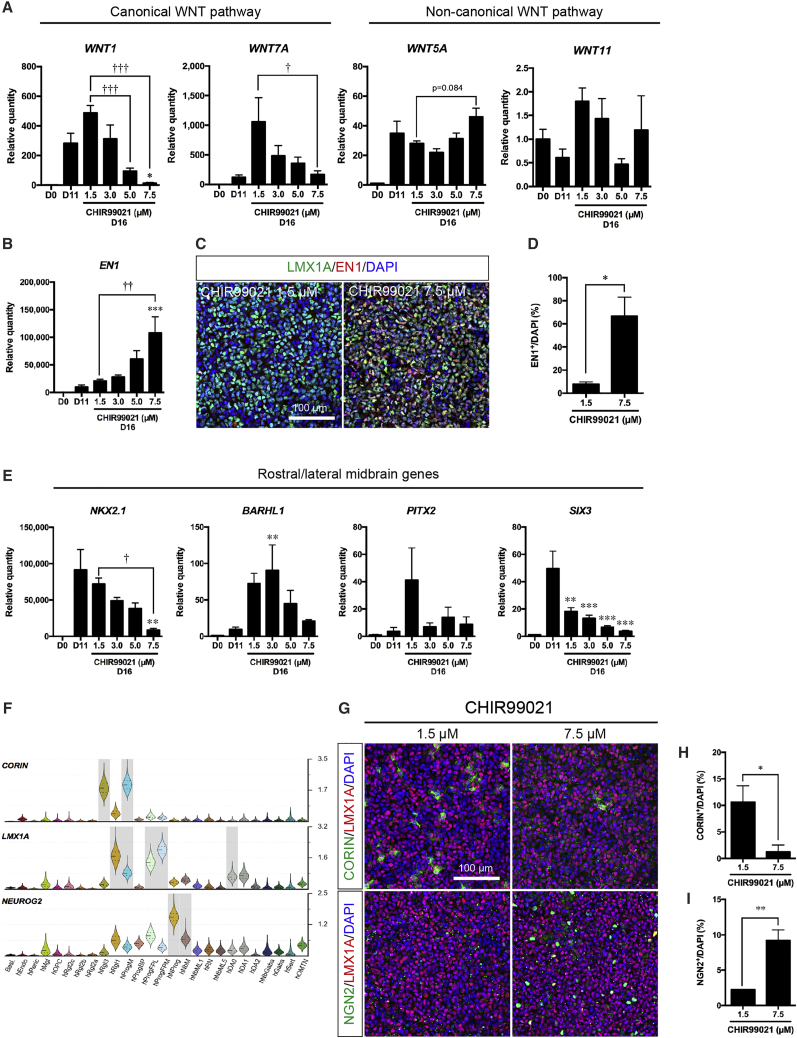
Analysis of floor plate patterning in hESC-derived neural progenitors at day 16 (A) qPCR analysis of canonical WNT pathway and non-canonical WNT pathway at day 16. ^∗^p < 0.05 versus D11; †p < 0.01, †††p < 0.001 versus 1.5 μM CHIR99021 (n = 6 independent experiments). (B) qPCR analysis of *EN1* at day 16. ^∗∗∗^p < 0.001 versus D11; ††p < 0.01 versus 1.5 μM CHIR99021 (n = 6 independent experiments). (C) Immunostaining of LMX1A^+^;EN1^+^ cells. Scale bar, 100 μm. (D) Quantification of EN1^+^/DAPI^+^ cells. ^∗^p < 0.05 versus 1.5 μM CHIR99021 (n = 3 independent experiments). (E) qPCR analysis of rostral and lateral midbrain markers at day 16. ^∗∗^p < 0.01, ^∗∗∗^p < 0.001 versus D11; †p < 0.05 versus 1.5 μM CHIR99021 (n = 6 independent experiments). (F) Violin plots of *CORIN, LMX1A*, and *NGN2* generated from scRNA-seq data of developing human ventral midbrain. (G) Immunostaining of CORIN^+^;LMX1A^+^ cells and NGN2^+^;LMX1A^+^ cells. Scale bar, 100 μm. (H and I) Quantification of CORIN^+^/DAPI^+^ cells (H) and NGN2^+^/DAPI^+^ cells (I). ^∗^p < 0.05, ^∗∗^p < 0.01 versus 1.5 μM CHIR99021 (n = 3 independent experiments).

It is known that mDA neurons originate in the caudal floor plate domain under the influence of Wnt1, a morphogen strongly expressed in the midbrain side of the MHB and in the two bands that define the lateral floor plate (Prakash et al., 2006; Wurst et al., 2001). In addition, the hindbrain side of the MHB expresses fibroblast growth factor 8b (FGF8b), a factor also known to induce mDA neurons (Ye et al., 1998). We therefore treated our cultures with both CHIR99021 and FGF8b in the presence of purmorphamine, to mimic the morphogens controlling the midbrain floor plate. To estimate the resulting strength of Wnt signaling during CHIR99021, purmorphamine, and FGF8b treatment, we examined the expression of endogenous canonical and non-canonical Wnts (Figure 2A). As expected, activation of canonical Wnt/β-catenin signaling with increasing concentrations of CHIR99021 downregulated the expression of *WNT1* and *WNT7A*, but did not affect the expression of *WNT5A* and *WNT11*. Notably, 7.5 μM CHIR99021 did not change the proportion of LMX1A^+^;FOXA2^+^/DAPI^+^ cells and LMX1A^+^;OTX2^+^/DAPI^+^ cells (Figures S2A–S2C), but increased the expression of *EN1* (Figure 2B) and the proportion of EN1^+/^DAPI^+^ cells compared with 1.5 μM CHIR99021 (Figures 2C and 2D). Moreover, rostral and/or lateral midbrain markers such as *NKX2.1, BARHL1, PITX2,* and *SIX3* decreased with the concentration of CHIR99021 (Figure 2E). Thus our results indicate that the combination of 7.5 μM CHIR99021 and FGF8b from day 11 to day 16 does not only effectively increase caudal midbrain gene expression, but decreases rostral and lateral gene expression.

We also investigated whether combined 7.5 μM CHIR99021 and FGF8b promotes lineage progression from proliferative to neurogenic progenitors. We therefore examined the presence of cells expressing *CORIN*, an early midbrain gene selectively expressed in Rgl3 and ProgM cell types, and *NGN2,* a gene selectively expressed in two cell types undergoing neurogenesis, NProg and NbM (Figure 2F). Analysis of hESCs treated with 7.5 μM CHIR99021 days 11 to 16 revealed a decrease in CORIN^+^ cells, and an increase in NGN2^+^ cells, compared with 1.5 μM CHIR99021 (Figures 2G–2I). In addition, we also observed a very low proportion of LMX1A^+^;CORIN+/DAPI^+^ ProgM cells and a higher proportion of LMX1A^+^;NGN2^+^/DAPI^+^ cells in three different hESC lines treated with 7.5 μM CHIR99021 (Figures S2D–S2H). Thus, our results indicate that treatment with 7.5 μM CHIR99021 and FGF8b promotes cell lineage progression toward neurogenesis.

### Promotion of neurogenesis in hESC-derived progenitors by LXR activation

**Figure 3:**
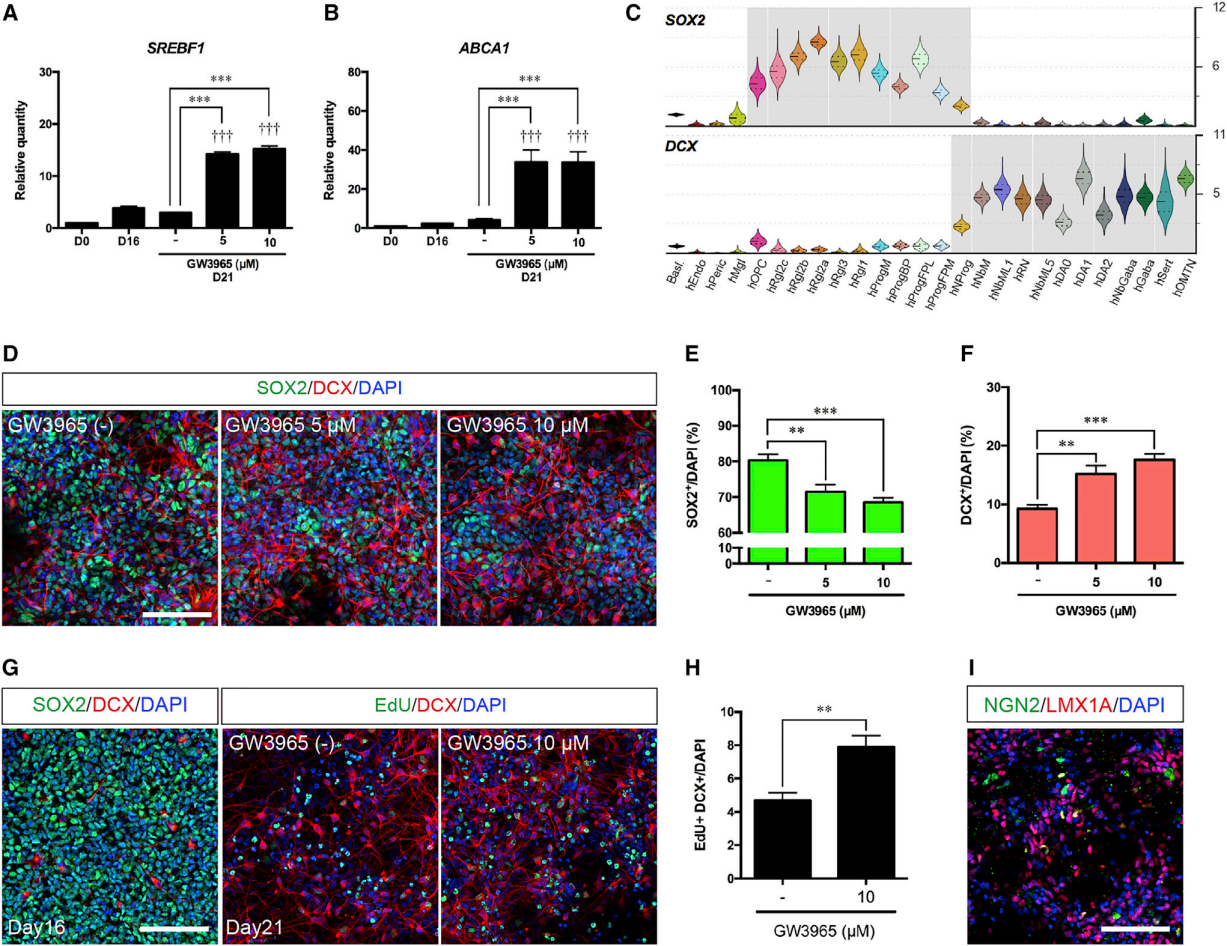
Analysis of hESC differentiation and neurogenesis at day 21 qPCR analysis of *SREBF1* (A) and *ABCA1* (B) at day 21. ^∗∗∗^ p < 0.001 versus GW3965(−) condition. ††† p < 0.001 versus day 16 (n = 6 independent experiments). (C) Violin plots of *SOX2* and *DCX* generated from scRNA-seq data of developing human ventral midbrain. (D) Immunostaining of SOX2^+^;DCX^+^ cells at day 21. Scale bar, 100 μm. (E and F) Quantification of SOX2^+^ cells (E) and DCX^+^ cells (F). ^∗∗^p < 0.01 and ^∗∗∗^p < 0.001 versus GW3965 (−) condition (n = 8 independent experiments). (G) Immunostaining of SOX2^+^;DCX^+^ cells at day 16 and EdU^+^; DCX^+^ cells at day 21, after GW3965 treatment (day 16–21). EdU pulse was performed for 4 h at day 16 and EdU detection was performed at day 21. Scale bar, 100 μm. (H) Quantification of EdU^+^;DCX^+^/DAPI^+^ cells at day 21. ^∗∗^p < 0.01 versus GW3965(−) condition (n = 6 independent experiments). (I) Immunostaining of LMX1A^+^ and NGN2^+^ cells at day 21. Scale bar, 100 μm.

On day 16, our cultures contained abundant LMX1A^+^, FOXA2^+^, and CORIN^−^ cells, indicating that most of the cells are ProgFPL and ProgFPM. On the other hand, we observed a growing number of NGN2^+^ cells within the LMX1A^+^ and FOXA2^+^ population, suggestive of an emerging NProg population. We thus examined whether neurogenesis could be enhanced by the synthetic LXR ligand, GW3965. Treatment with GW3965 (5–10 μM, days 16–21) upregulated the expression of the LXR target genes, *SREBF1* (Figure 3A) and *ABCA1* (Figure 3B), indicating effective activation of LXRs. In addition we monitored the expression of *SOX2,* a neural progenitor marker, and of doublecortin (*DCX*), a marker expressed during neurogenesis in NProg and in all postmitotic neuroblasts and neurons (Figure 3C). We found that 10 μM GW3965 significantly decreased the proportion of SOX2^+^ progenitors, and increased the proportion of DCX^+^ cells (Figures 3D–3F). Moreover, 5-ethynyl-2′-deoxyuridine (EdU) pulse-chase experiments revealed an increase in neurogenesis as shown by the increased proportion of EdU and DCX double-positive cells in the culture by 10 μM GW3965 at day 21 (Figures 3G and 3H). However, at this stage, immature cells including progenitors and NGN2^+^ cells were still present in the cultures (Figure 3I) and TH^+^ neurons had not yet emerged.

### Maturation of hESC-derived neurons by blocking of FGF signaling

**Figure 4:**
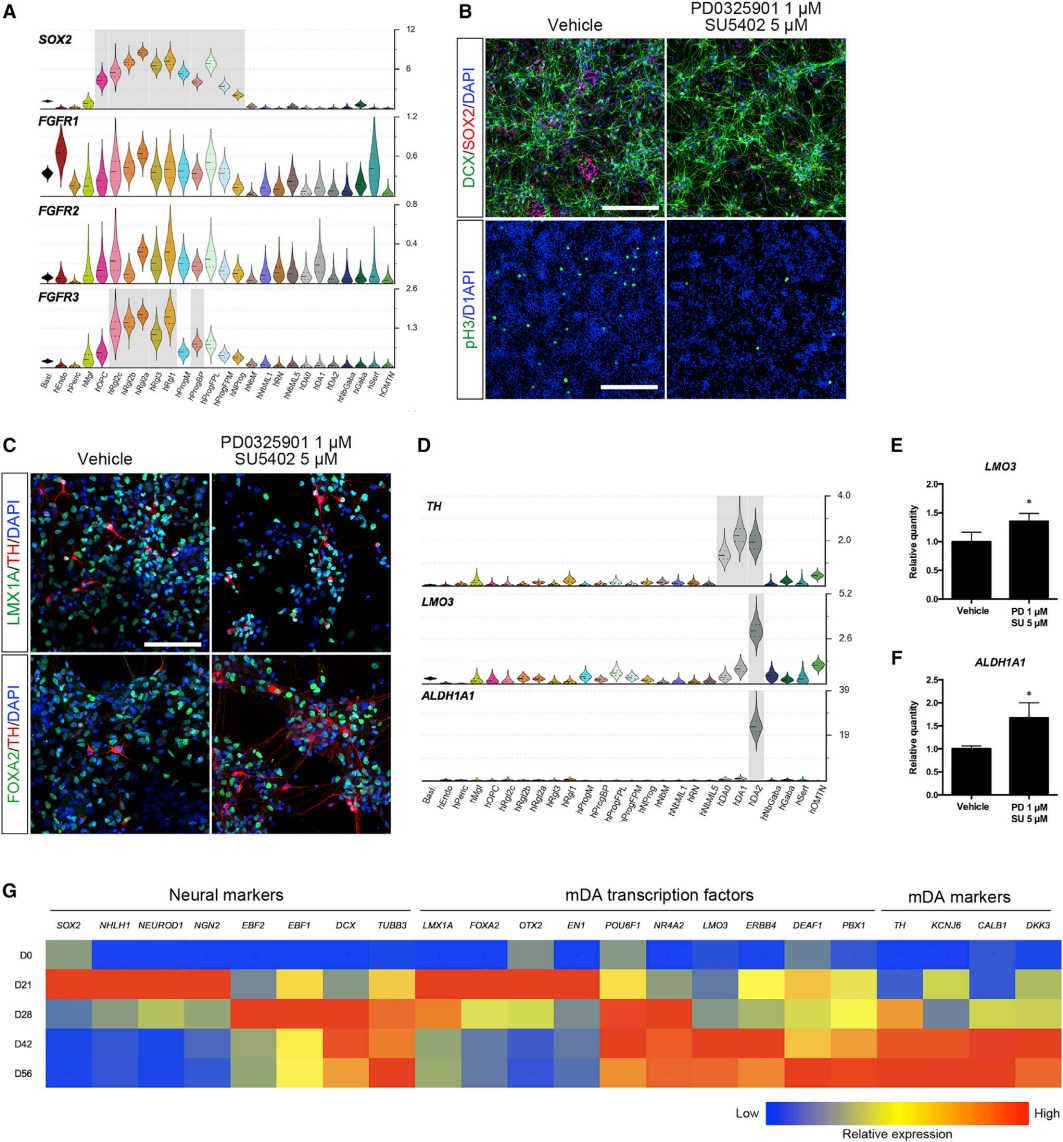
Analysis of postmitotic cells at day 28 and mDA neuron differentiation (A) Violin plots of *SOX2* and *FGFRs* generated from scRNA-seq data of developing human ventral midbrain. (B) Immunostaining of DCX^+^ and SOX2^+^ cells or pH3^+^ cells at day 28. Scale bars, 200 μm (upper panels) and 400 μm (lower panels). (C) Immunostaining of LMX1A^+^;TH^+^ cells and FOXA2^+^;TH^+^ cells at day 28. Scale bars, 100 μm. (D) Violin plots of *TH, LMO3*, and *ALDH1A1* generated from scRNA-seq data of developing human ventral midbrain. (E and F) qPCR analysis of *LMO3* (E) and *ALDH1A1* (F) at day 28. ^∗^p < 0.05 versus vehicle (n = 3 independent experiments). (G) Gene expression analysis during differentiation as assessed by qPCR. Values are color coded and normalized to the sample with highest expression for each gene (n = 2–3 independent experiments).

FGF receptors 1–3 are predominantly expressed in immature *SOX2*^+^ cell types in the developing human ventral midbrain, such as radial glia and progenitors (Figure 4A). Since FGF signaling is important to maintain and expand neural precursors (Elkabetz et al., 2008; Koch et al., 2009), we speculated that inhibition of FGF signaling may limit the growth of progenitors and promote their differentiation. To inhibit FGF signaling, cultures were treated from day 21 to day 28 with 1 μM PD0325901, a MEK/ERK pathway inhibitor, and 5 μM SU5402, an FGF receptor inhibitor. We found that treatment with PD0325901 and SU5402 drastically reduced SOX2^+^ cell clusters and the number of phospho-histone H3 (pH3)^+^ cells at day 28 (Figure 4B). At this stage, some cells exhibited neuronal morphology and expressed TH together with either LMX1A or FOXA2, suggesting the emergence of the dopaminergic DA0 neuronal population (Figure 4C). Markers identified at the single-cell level in SNc neurons and in embryonic dopaminergic neurons type 2 (DA2) (La Manno et al., 2016), such as *LMO3* and *ALDH1A1* (Figure 4D), were examined by qPCR and were found significantly increased at day 28 after treatment with PD0325901 and SU5402 (Figures 4E and 4F). These results indicate that blocking FGF signaling promotes the maturation of mDA neurons as shown by the increase in DA subtype marker expression. In agreement with these findings, a time-course analysis of gene expression by qPCR confirmed that the expression of progenitor markers (*SOX2*, *NEUROG2*, *LMX1A,* and *FOXA2*) peak at day 21 and decrease at day 28, while markers of postmitotic cells (*DCX* and *TUBB3*) and of mDA neurons (*NR4A2* and *TH*) peak at day 28 and remain stable thereafter (Figures S3A and S3B). Moreover, markers and transcription factors expressed at the single-cell level in mDA neurons (*TH, KNCJ6, CALB1, ERBB4,* and *PBX1*) or selectively in DA2 neurons (*LMO3, DEAF1, POU6F1*, and *DKK3*) (La Manno et al., 2016), increase at days 42 and 56, suggesting a stable generation of postmitotic mDA neurons (Figure 4G).

### Analysis of hESC-derived cell types by scRNA-seq

To further examine the quality of the hESC-derived midbrain cell types, we performed single-cell transcriptomics profiling of H9 and HS980 cells at days 0, 11, 16, 21, and 28 of differentiation. The quality of the cells was monitored by immunocytochemistry and found to be comparable for both cell lines at day 16 (Figures S4A and S4B).

**Figure 5:**
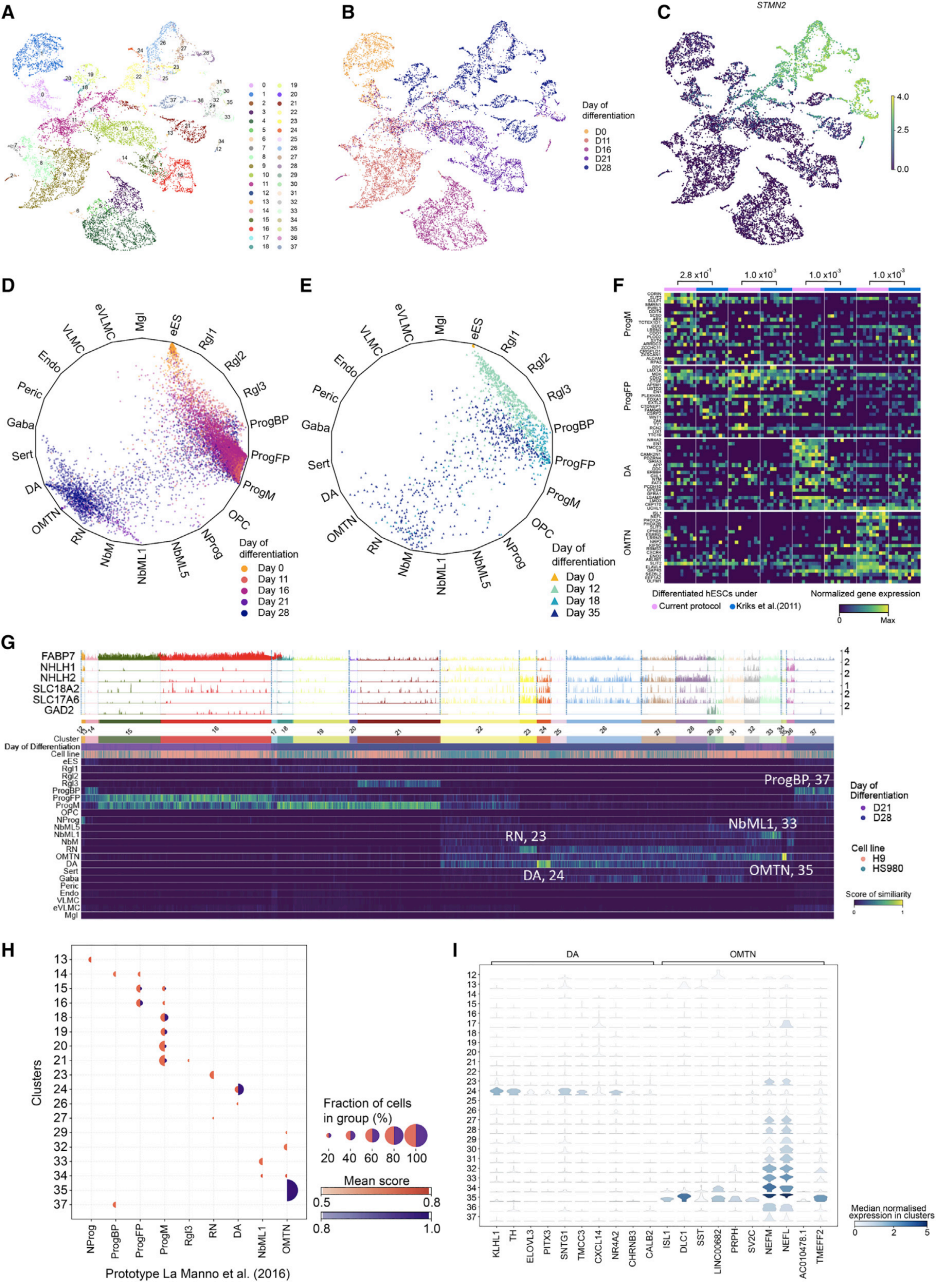
Analysis of hESC-derived cell types by scRNA-seq and logistic regression (A–C) UMAP projection of hESC-derived cells after quality filtering showing cells colored by their Louvain cluster’s membership (A), day of differentiation and analysis (B), and their log-library size normalized expression of *STMN2* (C). (D and E) Wheel plot showing hESC-derived midbrain cell types (dots) generated by the protocol developed in this study (D) or the protocol by Kriks et al. (2011) (E), compared by logistic regression to endogenous standards (wheel): human embryonic ventral midbrain cell types from La Manno et al. (2016) and vascular leptomeningeal cells from Marques et al. (2018). (F) Heatmap showing genes with highest coefficients from logistic regression for progenitor midline (ProgM), progenitor floor plate (ProgFP), dopaminergic neurons (DA), andoculomotor and trochlear nucleus (OMTNs). Log-library size normalized gene expression of top-similar hESCs to *in vivo* counterparts derived from current protocol and Kriks et al. (2011) are shown. Permutation test for each reference cell type was performed with H1: The sum of average expression of genes in the heatmap. Permutation was performed 1,000 times on normalized counts, p values are shown. (G) Track plot showing log-library size normalized gene expression of selected genes of cells at days 21 and 28 of differentiation (top). Heatmap showing similarities between differentiated cells and reference endogenous cell types from La Manno et al. (2016) and Marques et al. (2018), scoring using logistic regression (bottom). (H) Dot plot showing clusters from days 21 and 28 of differentiation with average similarity scores, as determined by logistic regression, between 0.5 and 0.79 (orange) or between 0.8 and 1.0 (purple). Only clusters with similarity scores >0.5 are shown. (I) Violin plot showing genes enriched in cluster 24 and 35. Log-library size normalized gene expression is shown.

After filtering, 11,681 high-quality cells were included in our analysis. The mean UMIs of cells at different time points of differentiation ranged between 6,753 and 15,482, and their mean transcripts between 2,490 and 3,931 (Figure S5A). Both H9 and HS980 contributed similar proportions of cells to the time points and clusters (Figures S5A and S5B). Dimensionality reduction and Louvain clustering with Cytograph revealed 38 clusters (Figure 5A). Cluster 1 was formed by undifferentiated hESCs at days 0 and 11, while clusters 0 and 2 to 11 were mainly contributed by cells from day 11 to 16, which have a higher proliferation index and are enriched in progenitor markers such as *SOX2* (Figures 5B, S5C and S5D). Clusters 12 to 37 were mainly contributed by days 21 and 28 and contained all the cells enriched in the expression of neuronal markers such as *STMN2* and *MYTL1* (Figures 5B, 5C and S5E).

### hESC derivatives are comparable to endogenous midbrain standards

Logistic regression was next used on scRNA-seq data to determine the probability of each of the hESC-derived cells being any of the endogenous human ventral midbrain tissue reference cell types as defined by La Manno et al. (2016) (Figures S5F–S5H). In addition, we also used a reference dataset of vascular leptomeningeal cells (VLMCs) (Marques et al., 2018) because this cell type was not previously found in the endogenous developing human ventral midbrain *in vivo* (La Manno et al., 2016), but has been detected in hESC-derived midbrain cultures (Tiklová et al., 2019). We found that our human development-based hESC differentiation protocol generates cells with low or extremely low probability of being cell types defined by non-ventral midbrain standards, such as hindbrain serotonin neurons or VLMCs, respectively (Figure 5D). Consistent with these data, double COL1A1^+^ and PDGFRA^+^ VLMCs were not detected at day 16. However, early CHIR99021 treatment (days 0–2) and removal of high CHIR99021 (7.5 μM) in the presence of FGF8b (days 11–16) induced the emergence of strongly double-positive COL1A1^+^ and PDGFRA^+^ cells (Figure S6). These results show that VLMCs are not present in our standard midbrain culture conditions, but they can emerge by premature CHIR99021 treatment in the absence of CHIR99021 boost.

We also found that cells generated in our cultures had high probability of being cell types defined by the *in vivo* human ventral midbrain standards (Figure 5D). The most abundant progenitor-like cell types were ProgM and progenitor floor plate (ProgFP), identified at day 16. Instead, postmitotic cell types such as oculomotor and trochlear neurons (OMTNs) were found at day 21, and DA at day 28. Notably, other neural cell types, such as oligodendrocyte progenitors or Rgl2, both found in the basal plate (La Manno et al., 2016), were not identified (Figure 5D). These findings indicate that the transcriptomic profiles of the hESC-derived cell types are comparable to those of cells in the most ventral aspect of the human midbrain *in vivo*.

### Improved quality and developmental dynamics of hESC-derived midbrain cell types compared with previous hESC differentiation conditions

Next we used our reference dataset to predict cell types from a previous scRNA-seq experiment (La Manno et al., 2016) in which H9 and HS401 hESC lines were differentiated for 12, 18, and 35 days into mDA neurons using the protocol by Kriks et al. (2011) (Figure 5E). Comparison of cells generated by the Kriks protocol to those generated by our new protocol, with improved developmental control, revealed four important differences. First, abundant basal plate progenitors but not midline progenitors were generated by the Kriks protocol (2011), while our new protocol generated more midline progenitors, reflecting an improved ventralization (Figures 5D and 5E). Second, a significant enrichment in the expression of genes defining endogenous human ProgM, such as *CORIN, SLIT2, SULF1,* and *ALCAM,* was found in the new compared with the old protocol (Figure 5F). Third, mDA neurons appeared at day 35 in the old protocol, but they were already abundant at day 28 in our new protocol (Figures 5D and 5E). Fourth, neurons of higher quality were found in the new protocol compared with the old one, as assessed by their similarity to OMTN and DA standards (dots near the vertices in the wheel/polygon plot, Figures 5D and 5E). Moreover, genes typically expressed by OMTNs (i.e., *ISL1, NEFL,* and *PHOX2A*), or DA neurons (i.e., *NR4A2, EN1,* and *TH*) were significantly enriched in cells generated by the new protocol compared with the old one (Figure 5F). Combined, these results suggest that our new protocol, compared with that by Kriks et al. (2011), generates cultures with improved cell composition and cell types of a quality closer to that in the human ventral midbrain *in vivo*.

### Correct developmental dynamics and high-quality cell types in hESC-derived cultures compared with endogenous midbrain standards

We first found that hESC-derived clusters enriched in day 21 and 28 cells (c12-37, Figures 5G and S5I) are similar to several cell types in the endogenous human ventral midbrain from week 6 to 11 (La Manno et al., 2016). At day 21, clusters expressing high levels of *FABP7* (c12-16) contained cells that resembled either neuronal progenitors expressing the pro-neural genes *NHLH1* and *NHLH2* (NProg, c12-13) or both endogenous floor plate and midline progenitors (ProgFP and ProgM, c15-16), or both floor plate or basal plate progenitors (c14). Notably, hESC-derived postmitotic cells expressing *STMN2* and *MYT1L* were also found at day 21 (clusters 29, 32–35, Figures 5A–5C). These clusters resembled mediolateral neuroblasts (NbML1, c33) or OMTNs (c35) (Figure 5G), both of which appear early in the human ventral midbrain *in vivo* (La Manno et al., 2016).

At day 28, we also observed a cluster with progenitors similar to endogenous basal plate progenitors (ProgBP, cluster 37), but most of the progenitor cells resembled midline progenitors (ProgM, clusters 18–21), with cluster 21 exhibiting additional features of Rgl3 identity (Figure 5G). We found cells expressing neurogenesis markers such as *NHLH1* and *NHLH2* (c22), and markers such as *SLC18A2* and/or *SLC17A6* (clusters 26–28 and 30–31), expressed by nascent mDA neurons (Kouwenhoven et al., 2020). Moreover, postmitotic day 28 cells resembled endogenous human week 6 to 11 red nucleus neurons (RN, c23) and dopaminergic neurons (hDA, c24). Thus, our results indicate that the most prominent progenitors derived from hESCs are the floor plate and midline progenitors at day 21, followed by the midline progenitor at day 28. Notably, neurons are generated with a developmental timing similar to that of their *in vivo* counterparts, with OMTNs being detected at week 7 *in vivo* and day 21 *in vitro*, followed by DA neurons at week 8 *in vivo* and day 28 *in vitro.*

Next, we scored the degree of similarity between hESC-derived cells and the endogenous standards. Cells reaching the highest degree of similarity (score 0.8–1) in ascending order were as follows: ProgFP (c15,16), ProgM (c18-21), mDA neurons (c24), and OMTNs (c35) (Figure 5H). Notably, 50% of the cells in c24 and 96% in c35 highly resembled endogenous mDA neurons (mean similarity score of 0.91) and OMTNs (0.98), respectively (Figure 5H). Accordingly, c35 was found selectively enriched in genes that define OMTNs such as *ISL1* and *DLC1*, while c24 was enriched in DA neuron genes, including *NR4A2*, *TH,* and *PITX3* (Figure 5I). Combined, these results indicate that our current differentiation protocol sequentially generates good-quality progenitors (floor plate followed by midline progenitors), and very high-quality neurons (OMTNs followed by mDA neurons), closely resembling endogenous midbrain development.

### hESC-derived mDA neurons become mature functional neurons

**Figure 6:**
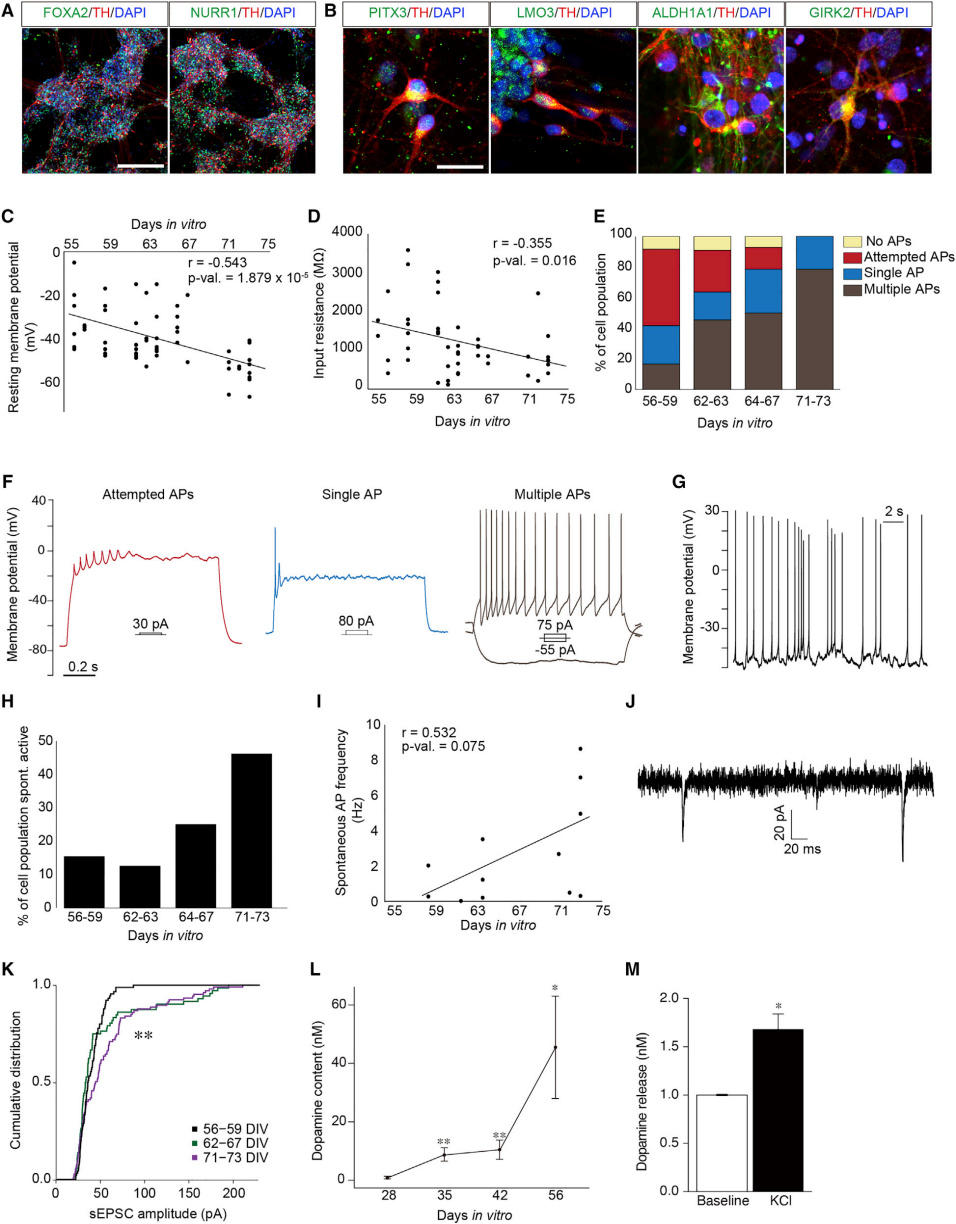
Maturation and functionality of the hESC-derived mDA neurons (A and B) Immunocytochemical staining of FOXA2^+^; TH^+^ cells as well as NURR1^+^; TH^+^ cells (A), as well as PITX3^+^; TH^+^ cells, LMO3^+^; TH^+^ cells, ALDH1A1^+^; TH^+^ cells, or GIRK2^+^/TH^+^ cells (B) at day 56. Scale bar, 200 μm (A) and 25 μm (B). (C–K) Electrophysiological analysis of cells from days 56 to 73. (C and D) Decrease of resting membrane potential (C) (n = 55 cells) and reduction in input resistance (D) (n = 46 cells) with increasing days *in vitro* (DIV). (E and F) Percentage of cell population exhibiting the ability to generate the different spiking types in response to square current pulses as seen in example traces (F) (56–59 DIV, n = 12 cells; 62–63 DIV, n = 11 cells; 64–67 DIV, n = 14 cells; and 71–73 DIV, n = 14 cells). (G) Example trace of a spontaneous active neuron. (H) Percentage of cells spontaneously spiking (56–59 DIV, n = 3 cells; 62–63 DIV, n = 8 cells; 64–67 DIV, n = 12 cells; and 71–73 DIV, n = 13 cells). (I) Spontaneous action potential (AP) frequency of cells that were spontaneously spiking (n = 12 cells). (J) Example trace of a cell receiving two spontaneous excitatory postsynaptic currents (sEPSCs). (K) Cumulative distribution of sEPSC amplitudes in each cell population (Kolmogorov-Smirnov test: 56–59 DIV versus 62–67 DIV p value = 0.243, 56–59 DIV versus 71–73 DIV p value = 7.97 × 10^−4^, 62–67 DIV versus 71–73 DIV p value = 9.67 × 10^−4^). (L and M) HPLC analysis of whole cell dopamine content from day 28–56 (L) and dopamine release at day 56 (M). ^∗^p < 0.05, ^∗∗^p < 0.01 (n = 4 independent experiments, error bars are SEM).

We first explored whether the mDA neurons generated *in vitro* at day 28 can develop into mature mDA neurons. Analysis of marker expression at day 56 revealed that TH^+^ neurons are FOXA2^+^ and NURR1^+^ (Figure 6A). Moreover, some of the TH^+^ neurons were positive for markers associated with mature mDA neurons, such as PITX3^+^, LMO3^+^, ALDH1A1^+^, or GIRK2^+^ (Figure 6B), indicating that hESC-derived mDA neurons adopt mature midbrain phenotypes.

We next performed electrophysiological recordings to examine whether these cells can mature into functional neurons. Analysis of the membrane resting potential and input resistance revealed a progressive decrease of these two parameters from day 56 until day 73, indicating further maturation during this period (Figures 6C and 6D). Similarly, current-clamp recordings from day 56 to 73 revealed improved firing capacity upon current injections (Figures 6E and 6F). Notably, the proportion of neurons responding with multiple or single action potentials increased from 42% at days 56 to 59, to close to 100% at days 71 to 73. Moreover, the proportion of neurons exhibiting spontaneous electrical activity and action potentials also increased from day 56 to 73 (Figures 6G–6I). In addition, the patched cells received spontaneous excitatory postsynaptic currents (sEPSCs) from days 56 to 73 (Figures 6J and 6K), suggesting the establishment of synaptic connections. Combined, these results indicate that our human development-based differentiation protocol gives rise to neurons that progressively mature in culture and become electrophysiologically active by days 71 to 73. Finally, we examined whether these neurons also acquire the capacity to synthesize and release the neurotransmitter dopamine (Figures 6L and 6M). High-performance liquid chromatography (HPLC) revealed very low levels of dopamine content at day 28, an increase at days 35 and 42, and high levels at day 56, whereas dopamine release was only detected at day 56. Thus, the results above show that hESC-derived mDA neurons progressively acquire functional properties of mature mDA neurons *in vitro*.

## Discussion

In this study, we address the challenge of achieving hESC-derived products with sufficient molecular definition and similarity to endogenous standards with the goal of enabling their future development for drug development and cell replacement therapy. We show that by modulating different pathways in a time-controlled manner in hESCs, it is possible to reproduce key aspects of the developmental dynamics of the ventral midbrain and improve midbrain patterning as well as mDA neurogenesis and differentiation. ScRNA-seq analysis revealed sequential generation of hESC-derived ventral midbrain progenitors and neurons with single-cell transcriptomics profiles similar to those found in the endogenous human midbrain. Moreover, these profiles were of higher quality than those obtained with a previous mDA differentiation protocol. In addition, we find that hESC-derived mDA neurons can mature and become functional *in vitro*. Indeed, mDA neurons appear by day 28, express mature mDA markers by days 42 to 56, acquire the capacity to release dopamine by day 56, and become electrophysiologically active neurons by day 73.

Our work additionally defines the function of a number of key developmental pathways, which have not been previously examined or used to differentiate hESCs into mDA neurons. Factors such as full-length LN511, the morphogen WNT5A, and the combination of FGF8b and high CHIR99021 (7.5 μM) were found to affect anterior-posterior patterning. For instance, LN511 and WNT5A decreased the expression of hindbrain genes such as *GBX2* and *HOXA2* at day 11. Moreover, WNT5A as well as high CHIR99021 combined with FGF8b, decreased the expression of anterior and lateral genes (*FOXG1, BARHL1, PITX2, SIX3,* and *NKX2.1*), and increased expression of midbrain genes (*LMX1A* and *EN1*) at days 11 and 16. In addition, cell types such as VLMCs, absent in our developmental standards and in other hESC-derived ventral midbrain cultures (Kim et al., 2021), were not detected during mDA differentiation. Interestingly, a modified protocol involving early CHIR99021 administration (day 0–11) and FGF8 treatment in the absence of CHIR99021 boost (day 9–16) gave rise to VLMCs, suggesting this cell type can emerge in specific culture conditions, as previously reported (Tiklová et al., 2020).

We also found that sequential administration of the small molecules CHIR99021 (7.5 μM) and GW3965, to activate Wnt/β-catenin and LXR signaling, respectively, control different aspects of neurogenesis. Indeed, high CHIR99021 increased the number of NGN2^+^ cells at day 16, a gene required for mDA neurogenesis (Kele et al., 2006), while GW3965 improved neurogenesis (EdU^+^; DCX^+^ cells) and reduced the number of SOX2^+^ cells at day 21. In addition, we found that treatment with the FGF receptor inhibitor, SU5402, and the MEK/ERK inhibitor, PD0325901, further reduced proliferation and SOX2^+^ cells at day 28. At this stage, abundant TH^+^ neurons expressed midbrain markers such as LMX1A, FOXA2, NURR1, PITX3, LMO3, ALDH1A1, and GIRK2 (KCNJ6), indicating efficient mDA neurogenesis.

Single-cell transcriptomics allowed us to perform a detailed analysis of the molecular cell types generated in our hESC cultures compared with endogenous human ventral midbrain standards (La Manno et al., 2016). This comparison enabled us to define the identity of the cell types generated *in vitro* as well as their quality and their developmental dynamics. Analysis of hESC-derived cultures revealed the presence of good-quality progenitors, which followed a temporal sequence of events similar to that found *in vivo*. The floor plate progenitor (c14-16) was enriched at day 21 and was nearly absent at day 28, whereas the midline progenitor (c18-21) was present at day 21 and is abundant at day 28. Notably we found that the identity of progenitors at day 21 was less well defined than at day 28. Indeed, day 21 progenitor clusters contained two types of progenitors, floor plate and basal plate (c14) or floor plate and midline (c15, 16). In addition, some progenitors in these clusters partially shared the two identities, suggesting the presence of cell transitions or earlier progenitors that are not present in week 6 to 11 developmental standards and are thus only partially recognized. Instead, day 28 clusters contained only one type of progenitor, either midline (c18-21) or basal plate progenitors (c37), suggesting that they have refined their identities and are then recognized by our developmental standards.

As expected by the presence of basal plate, floor plate, and midline progenitors, our cultures give rise to diverse postmitotic cell types found in the endogenous human ventral midbrain during weeks 6 to 11. Interestingly these cells also emerge following a specific developmental sequence of events, with mediolateral neuroblast 1 (NbML1, c33), and OMTNs (c35) emerging at day 21, followed by red nucleus (c23) and mDA neurons (c24) at day 28. Notably, the quality of mDA neurons was very high already at day 28, with 50% of the hESC-derived mDA neurons showing a transcriptome 91% similar to that of endogenous embryonic human mDA neurons. These results show that our human development-based differentiation protocol, by improving developmental control of hESCs during mDA differentiation, recapitulates multiple aspects of human ventral midbrain development, including the temporal axis and the generation of high-quality prototypical cell types as defined by scRNA-seq analysis. We therefore suggest the current differentiation paradigm may be useful to model and study human mDA neuron development and functionality *in vitro*. Moreover, since human ventral midbrain tissue has been successfully used for cell replacement therapy in PD patients (Kefalopoulou et al., 2014; Li et al., 2016; Lindvall et al., 1990), and hPSC-derived DA progenitors are currently being used in clinical trials for PD cell replacement therapy (Barker et al., 2017; Doi et al., 2020; Kim et al., 2021; Piao et al., 2021; Schweitzer et al., 2020; Tao et al., 2021), we suggest our differentiation paradigm may also be useful for this type of application. We envision that strategies aiming at generating or selecting molecularly defined cell types, such as the progenitor of the dopaminergic neuron subtype mainly affected by disease, the SOX6_AGTR1 subpopulation (Kamath et al., 2022), may enable highly precise and safe cell replacement therapy for PD.

In the near future, we expect that hESC-derived preparations destined for cell replacement therapy will be routinely examined at the single-cell level in order to control for cell composition and quality. In this context, our work represents a first attempt to compare cell preparations with endogenous standards, but more work will be needed to improve the resolution of the single-cell analysis. This will involve (1) improving the definition of endogenous human midbrain standards, with more time points, deeper coverage, and multimodal single-cell data; (2) correlating cell composition and quality at the single-cell level *in vitro* with the preclinical and clinical performance of the grafts; and (3) developing new computational methods and tools to integrate multiple levels of information and precisely compare hPSC-derived cell types with endogenous standards and functionality *in vitro* and *in vivo*. Ultimately, we should be able to design and develop hPSC preparations with the desired cell composition, single-cell quality, and functionality for specific and precise *in vitro* and *in vivo* applications.

## Experimental procedures

### Resource availability

#### Corresponding author

Ernest Arenas, ernest.arenas@ki.se

#### Materials availability

This study did not generate any unique reagents.

#### Data and code availability

RNA sequencing datasets are available at the European Genome-Phenome Archive (EGA), study ID: EGAS00001006313.

### Undifferentiated human ESC culture

Human ESC lines H9 (Thomson et al., 1998), HS401, HS975, and HS980 (Rodin et al., 2014) were maintained on LN521 (BioLamina)-coated dishes in NutriStem XF hESC medium (Biological Industries). Cells were passaged with TrypLE Select (Thermo Fisher Scientific) every 4 to 6 days, and were re-plated at a density of 50,000 to 100,000 cells/cm^2^ in medium supplemented with 10 μM Y27632 (Tocris) for the first 24 h.

### Additional methods

Please see the supplemental experimental procedures.

## Author contributions

K.Nishimura, S.Y., and E.A. designed the project. K.Nishimura performed the experiments in Figures 1, 2, 3, 4, 6A, 6B, S1, S2, S3, and S4. S.Y. and E.S.A. performed additional DA differentiation experiments (Figures 5, 6, and S6) with support from C.S. and G.L. S.Y. and L.H. performed scRNA-seq with support from S.L. K.L. performed the bioinformatics analysis in Figures 5 and S5. K.Nikouei, S.G., and J.H.L. contributed to the electrophysiological analysis in Figures 6C–6K and interpretation. W.P. and P.S. contributed to the analysis of dopamine content and release in Figures 6L and 6M and interpretation. E.A. supervised the project, co-wrote the manuscript with K.Nishimura, S.Y., and K.L. All authors reviewed and approved the manuscript.

## Supplemental information

Document S1. Figures S1–S6, Table S1, and supplemental experimental procedures

Document S2. Article plus supplemental information
