# Supplementary material for: Single-cell transcriptomics reveals correct developmental dynamics and high-quality midbrain cell types by improved hESC differentiation
Source: Stem Cell Reports. 2022 Nov 17;18(1):337–53. doi: 10.1016/j.stemcr.2022.10.016 (PMC9860082; doi:10.1016/j.stemcr.2022.10.016)
Supplement: Document S1. Figures S1–S6, Table S1, and supplemental experimental procedures [file mmc1.pdf]

Stem Cell Reports, Volume *18*

Supplemental Information

Single-cell transcriptomics reveals correct developmental dynamics and high-quality midbrain cell types by improved hESC differentiation

Kaneyasu Nishimura, Shanzheng Yang, Ka Wai Lee, Emilía Sif Ásgrímsdóttir, Kasra Nikouei, Wojciech Paslawski, Sabine Gnodde, Guochang Lyu, Lijuan Hu, Carmen Saltó, Per Svenningsson, Jens Hjerling-Leffler, Sten Linnarsson, and Ernest Arenas

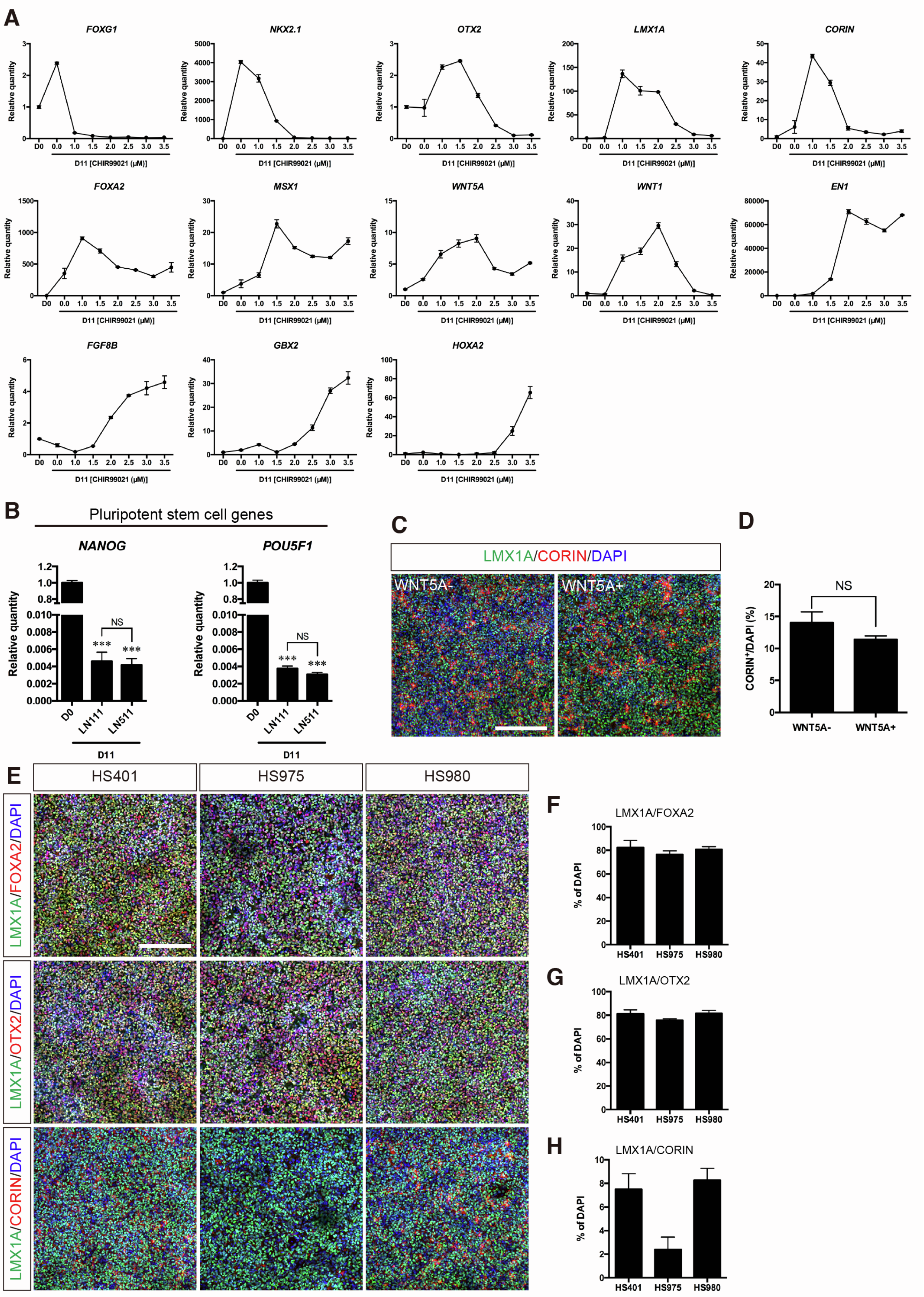
A
FOXG1
NKX2.1
OTX2
LMX1A
CORIN
FOXA2
MSX1
WNT5A
WNT1
EN1
FGF8B
GBX2
HOXA2
Relative quantity
D11 [CHIR99021 (µM)]
B
Pluripotent stem cell genes
NANOG
POU5F1
D0
LN111
LN511
D11
NS
***
C
LMX1A/CORIN/DAPI
WNT5A-
WNT5A+
D
CORIN+/DAPI (%)
E
HS401
HS975
HS980
LMX1A/FOXA2/DAPI
LMX1A/OTX2/DAPI
LMX1A/CORIN/DAPI
F
LMX1A/FOXA2
G
LMX1A/OTX2
H
LMX1A/CORIN
% of DAPI

Figure S1. Patterning of hESCs into midbrain floor plate progenitors, related to Figure 1
(**A**) qPCR analysis of differentiated cells according to CHIR99021 concentration at day 11 (n = 3 independent experiments). (**B**) qPCR analysis of *NANOG* and *POU5F1* of differentiating cells on LN111 and LN511. ***p < 0.001 vs. D0. NS, not significant (n = 6 independent experiments). (**C**) Immunostaining of $LMX1A^+$;$CORIN^+$ cells at day 11. Scale bar, 200 μm. (**D**) Quantification of $CORIN^+$ cells at day 11. NS, not significant (n = 3 independent experiments). (**E**) Immunostaining of $LMX1A^+$;$FOXA2^+$ cells, $LMX1A^+$;$OTX2^+$ cells and $LMX1A^+$;$CORIN^+$ cells in HS401, HS975 and HS980 hESC lines at day 11. Scale bar, 200 μm. (**F**-**H**) Quantification of $LMX1A^+$;$FOXA2^+$/$DAPI^+$ cells (**F**), $LMX1A^+$;$OTX2^+$/$DAPI^+$ cells (**G**) and $LMX1A^+$;$CORIN^+$/$DAPI^+$ cells (**H**) at day 11 in the WNT5A+ condition (n = 3 independent experiments).

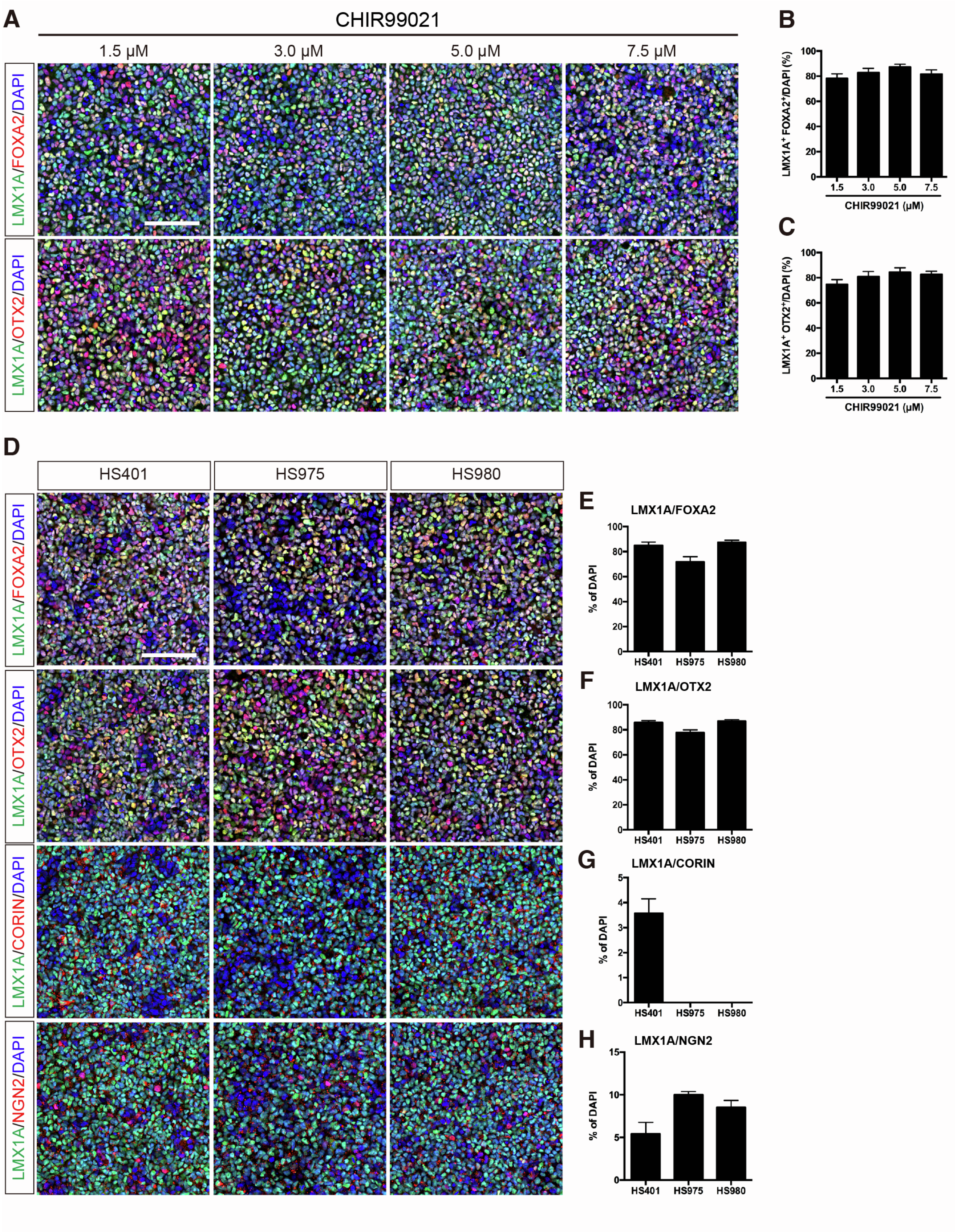

Figure S2. Characterization of DA progenitors derived from three different hESCs, related to Figure 2. (**A**) Immunostaining of LMX1A$^+$;FOXA2$^+$ cells and LMX1A$^+$;OTX2$^+$ cells at day 16, after exposure of HS980 cells to different concentrations of CHIR99021. Scale bar, 100 μm. (**B** and **C**) Quantification of LMX1A$^+$;FOXA2$^+$/DAPI$^+$ cells (**B**), LMX1A$^+$;OTX2$^+$/DAPI$^+$

cells (**C**) at day 16 (n = 3 independent experiments). (**D**) Immunostaining of LMX1A$^+$;FOXA2$^+$ cells, LMX1A$^+$;OTX2$^+$ cells, LMX1A$^+$;CORIN$^+$ cells and LMX1A$^+$;NGN2$^+$ cells in HS401, HS975 and HS980 hESC lines at day 16 of differentiation with 7.5 μM CHIR99021. Scale bar, 100 μm. (**E**-**H**) Quantification of LMX1A$^+$;FOXA2$^+$/DAPI$^+$ cells (**E**), LMX1A$^+$;OTX2$^+$/DAPI$^+$ cells (**F**), LMX1A$^+$;CORIN$^+$/DAPI$^+$ cells (**G**) and LMX1A$^+$;NGN2$^+$/DAPI$^+$ cells (**H**) at day 16 (n = 3 independent experiments).

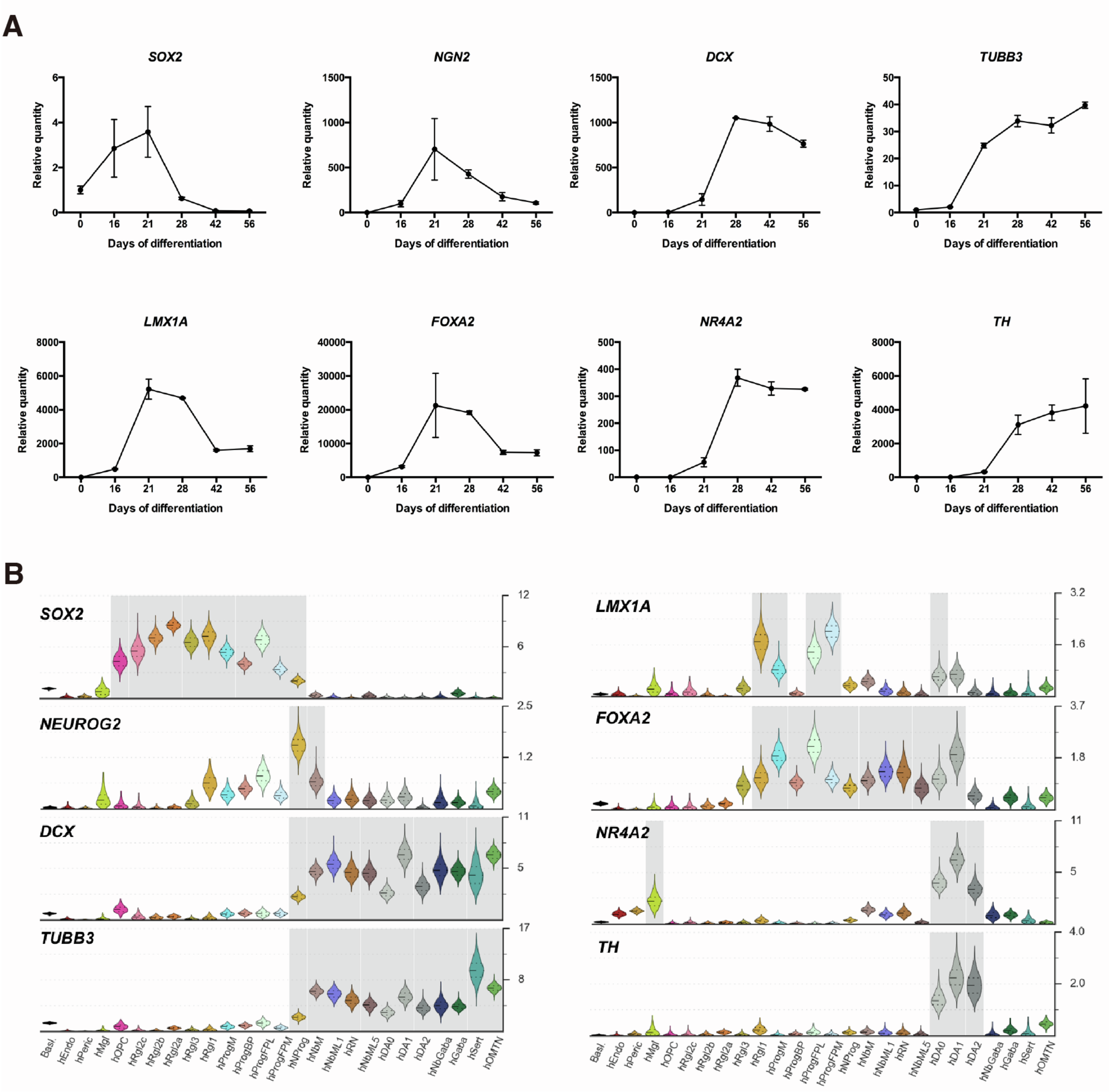

Figure S3. Time course of gene expression during hESC differentiation into mDA neurons, related to Figure 4. (**A**) qPCR analysis of midbrain progenitor and mDA neuron markers during hESC differentiation (n = 3 independent experiments) (**B**). Violin plots generated from scRNA-seq data for genes expressed in the developing human ventral midbrain *in vivo*.

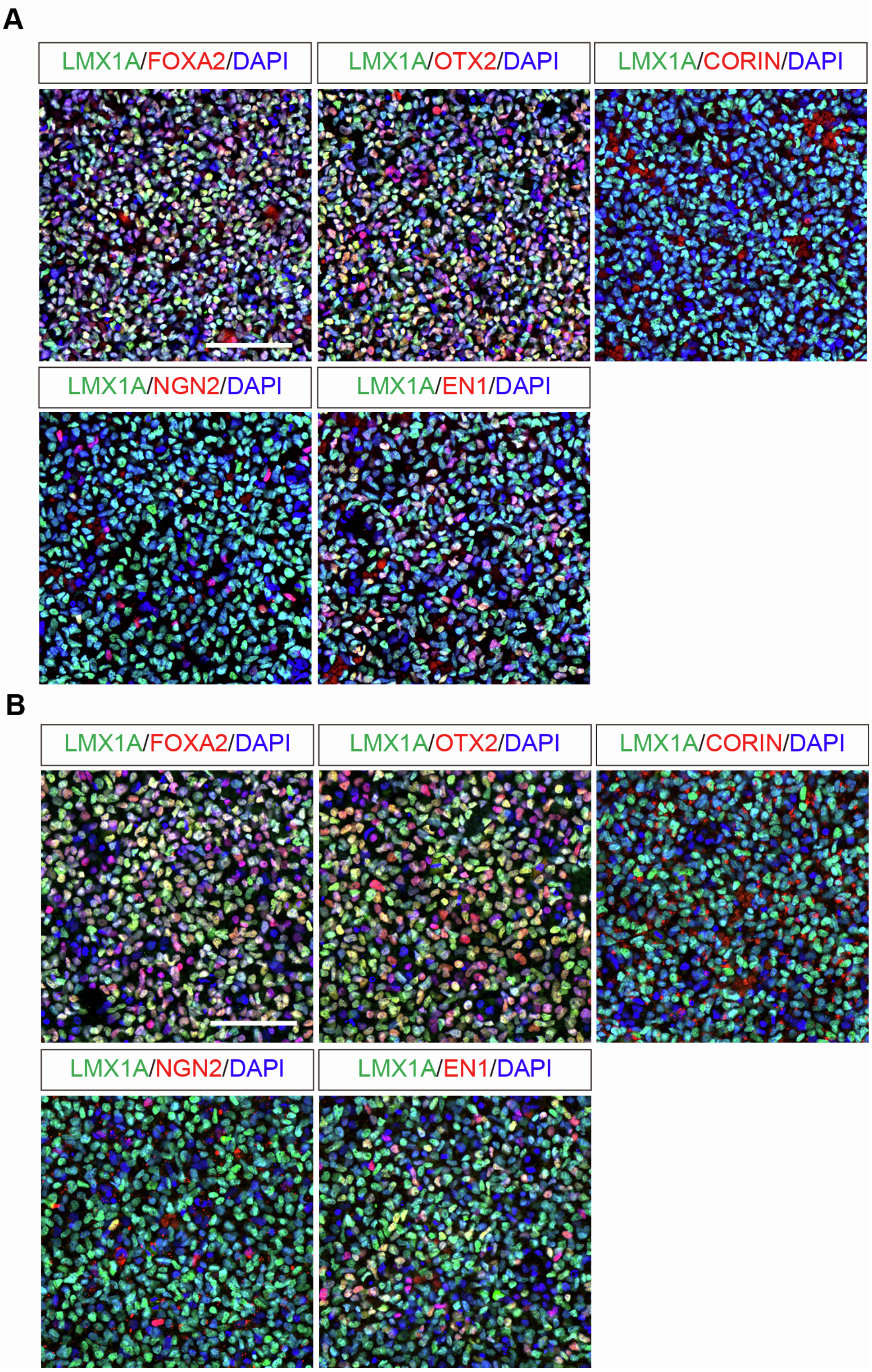

Figure S4. Validation of hESC-derived mDA progenitors by immunocytochemistry prior to scRNA-seq analysis, related to Figure 5. Immunofluorescence of hESC-derived mDA progenitors derived from H9 (**A**) and HS980 (**B**) hESCs at day 16. Scale bar, 100 µm.

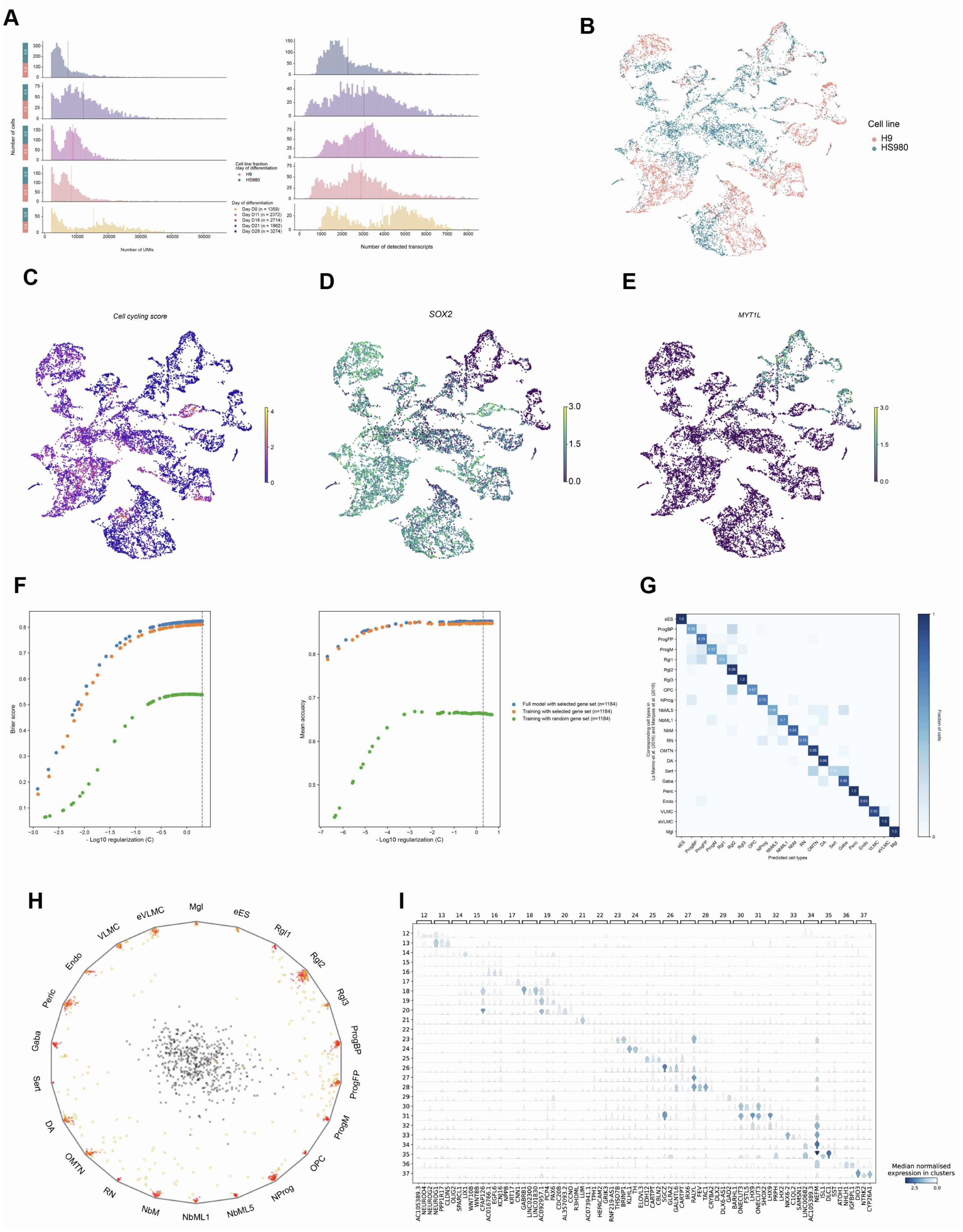

Figure S5. Analysis of hESCs-derived midbrain cell types by scRNA-seq, related to Figure 5. (**A**) Histogram showing the distribution of UMIs (right panel) and detected transcripts (left panel) per cell and day of differentiation. Bars show the fractions of cells generated by

each of the two cell lines (H9 or HS980) at the indicated day of differentiation. (**B-E**) UMAP projection of hESCs-derived cells as in Figure 5A, showing cells coloured by cell line of origin (**B**) cell cycle score (**C**) and their log-library size normalised gene expression of *SOX2* (**D**) and *MYT1L* (**E**). (**F**) Left: Mean Brier score per trial. Right: Mean accuracy score per trial. Dotted line indicates the parameter used for the final model (C = 1.99). (**G**) Corresponding between predicted cell types and reference cell types of 20% of the reference data after using 80% of the reference data for optimizing regularization strength in logistic regression. (**H**) Individual cells from training set (Red circles, 80% of the reference data), test set (Yellow circles, 20% of the reference data), and negative control (Black crosses, 20% of the reference data with random permutation of the selected gene set) plotted on a wheel plot. (**I**) Violin plot showing genes enriched in clusters from day 21 and 28 of differentiation. Log-library size normalized gene expression is shown.

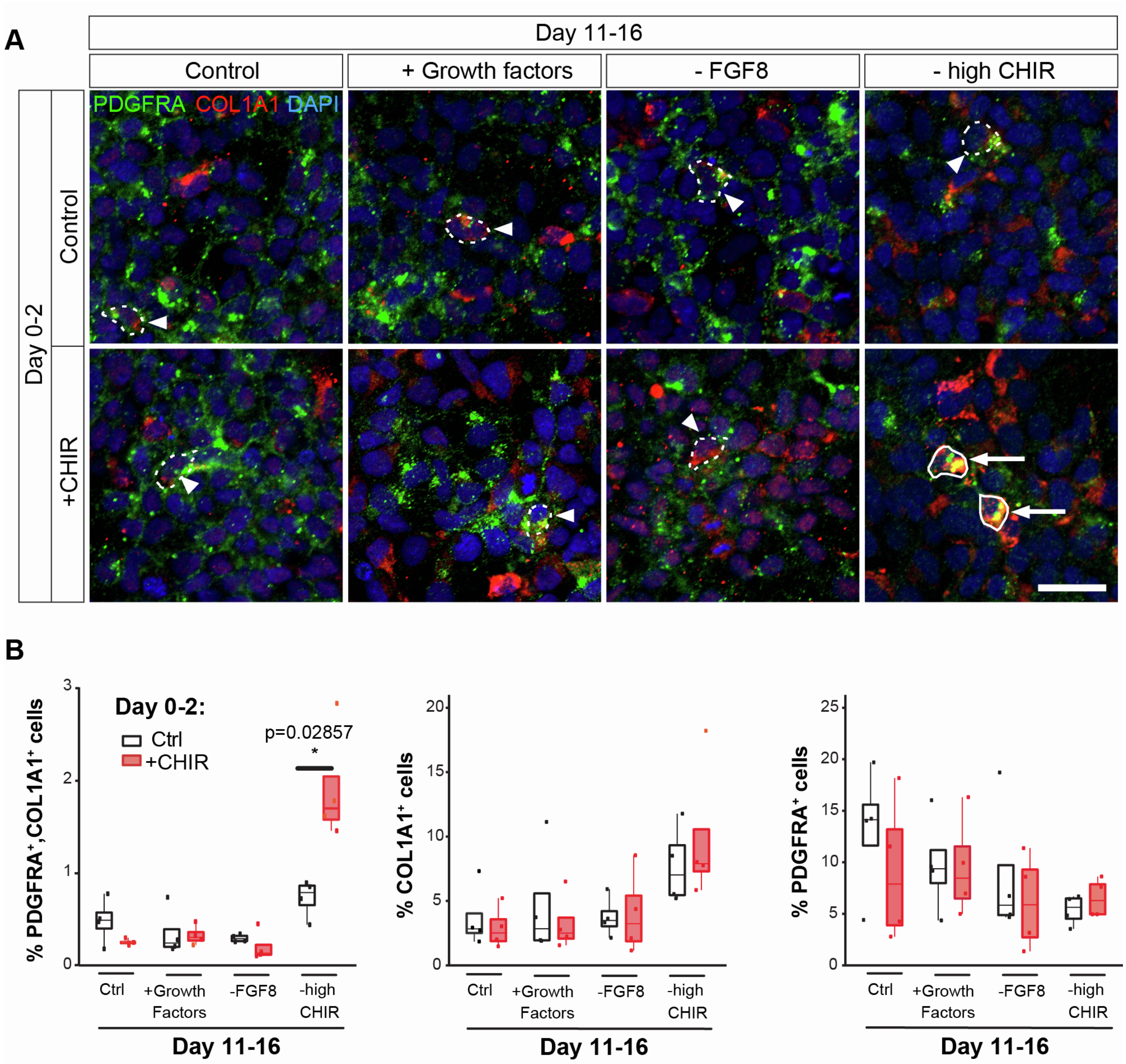

Figure S6. VLMCs emerge after abnormal patterning with CHIR99021, related to Figure 5. (**A**) Fluorescence immunocytochemistry staining for PDGFRA and COL1A1 showing that VLMCs, defined by the co-localization of PDGFRA and COL1A1, are not present in our standard culture conditions (control) or after adding growth factors or removing FGF8b at day 16. Background levels of co-localization (very few pixels in the same cell) were found in few cells (dotted line and arrowhead). However, abnormal Wnt patterning with CHIR99021 too early (Day 0-2) and too short (until day 11) gives rise to clear double+ cells that we identify as VLMCs (encircled cells pointed by arrows). (**B**) Percentage of $PDGFRA^{+}$, $COL1A1^{+}$ and $PDGFRA^{+}$;$COL1A1^{+}$ cells out of the total cells (DAPI) in different conditions. Values under 1% $PDGFRA^{+}$;$COL1A1^{+}$ cells resulted from background levels of staining (few pixels). Mann–Whitney U-test was used for pair comparison. Scale bar, 25 µm.

Supplemental experimental procedures

Differentiation protocol into mDA neurons

hESCs were seeded at a density of 500,000 cells/cm^2 on LN511 (BioLamina)-coated dishes in NutriStem XF hESC medium with 10 μM Y27632. Cells were first differentiated in TeSR-E6 medium (Stem Cell Technologies) supplemented with nonessential amino acids (Thermo Fisher Scientific), L-glutamine (Thermo Fisher Scientific), and 0.1 mM 2-mercaptoethanol (Gibco). At day 0, 200 nM LDN193189 (Stemgent), 10 μM SB431542 (Tocris) and 10 μM Y27632 were added. 2 μM purmorphamine (Stemgent) was incorporated at day 1, and 1.5 μM CHIR99021 (Sigma) at day 3. 10 μM Y27632 was removed from culture medium on day 3. The medium was gradually changed to neurobasal medium (Thermo Fisher Scientific) with B27 supplement (Thermo Fisher Scientific) and 2 mM L-glutamine from day 5 to day 11. SB431542 was removed at day 7 and 100 ng/mL Wnt5A (R&D Systems) was added. 100 ng/mL FGF8b (Peprotech) was incorporated from day 9-16. On day 11, the cells were dissociated into single cells and were re-plated on LN511-coated dish at a density of 500,000 cells/cm^2 in neurobasal medium with B27 supplement and 2 mM L-glutamine and treated with 100 ng/mL FGF8b, 7.5 μM CHIR99021 from day 11 to day 16. 10 μM Y27632 was in medium the first 24 hr after re-plating. On day 16, the cells were dissociated into single cells and were re-plated on LN511-coated dish at a density of 700,000 cells/cm^2 in neurobasal medium with B27 supplement and 2 mM L-glutamine. 10 μM GW3965 (Sigma), 10 μM DAPT (Sigma), 20 ng/mL brain-derived neurotrophic factor (BDNF) (R&D Systems), 200 μM ascorbic acid (Sigma) were supplemented from day 16 to day 21. 10 μM Y27632 was in medium the first 24 hr after re-plating. At day 21, 10 μM GW3965 was removed and the cultures received 10 ng/mL glial-cell derived neurotrophic factor (GDNF) (R&D Systems), 500 μM dbcAMP (Sigma) and 1 ng/mL transforming growth factor (TGF)β3 (R&D Systems) until the end of the culture. 1 μM PD0325901 (Sigma) and 5 μM SU5402 (Sigma) was added day 21-28 (**Figure 1A**).

Derivation of VLMCs from hESCs

Different variables in our mDA differentiation protocol were modified to examine whether VLMCs can be generated from hESCs when differentiated in culture conditions described in other protocols in the literature (Doi et al., 2014; Kim et al., 2021; Tiklová et al., 2019). To this end we compared administration of CHIR99021 (1.0 μM) at day 0 vs day 2 (1.5 μM, our protocol), and combined these two variables together with FGF8b (100 ng/mL in our protocol vs no treatment), CHIR99021 at day 11 to day 15 (7.5 μM in our protocol vs no treatment), as well as administration of growth factors and small molecules from day 11 to 15 (20 ng/mL BDNF, 20 ng/mL GDNF, 500 μM dbcAMP and 200 μM AA). Cells were re-plated at day 16 in neurobasal medium with B27 supplement, 2 mM L-glutamine, 100 ng/mL FGF8b, 20 ng/mL BDNF, 20 ng/mL GDNF, 10 μM DAPT, 500 μM dbcAMP, 200 μM AA and 10 μM Y27632. Analysis was performed by immunocytofluorescence at day 17.

cDNA synthesis and qPCR

Total RNA was extracted from the cells using RNeasy Plus Kit (Qiagen). 500 ng-1 μg total RNA was used for reverse transcription by a Super Script II First strand synthesis system with random primer (Thermo Fisher Scientific). qPCR was performed by using StepOne detection system (Applied Biosystems). Data analysis is based on dCt method with normalization of the raw data to *GAPDH* genes. Primer sequences were listed in Supplementary Table 1.

Fluorescent immunocytochemistry

The cells were fixed by 4% paraformaldehyde (PFA) for 30 min at 4˚C. The samples were then pre-incubated by 5% donkey serum in phosphate buffered saline containing 0.3% Triton X-100 (PBST) for 1 hr. The samples were incubated with primary antibodies at 4˚C overnight. The samples were incubated with either Alexa488 or Alexa555 or Alexa647-conjugated secondary antibodies (Thermo Fisher Scientific) for 30 min and then were incubated with 4',6-diamidino-2-phenylindole (DAPI) for 15 min. The primary antibodies were used as follows: ALDH1A1 (rabbit, 1:1,000, Abcam, ab23375), COL1A1 (sheep, 1:200, R&D Systems, AF6220), CORIN (rat, 1:1,000, R&D Systems, MAB2209), DCX (goat, 1:500, SantaCruz, sc-8066), EN1 (mouse 1:50, DSHB, 4G11), FOXA2 (goat, 1:500, R&D Systems, AF2400), GIRK2 (rabbit, 1:400, Alomone, APC006), LMX1A (rabbit, 1:4,000, Millipore, AB10533), LMO3 (goat, 1:200, SantaCruz, sc-82647), MAP2 (mouse 1:1,000, Sigma, M4403), NGN2 (goat, 1:200, SantaCruz, sc-19233), NURR1 (rabbit, 1:500, SantaCruz, sc-990), OTX2 (goat, 1:1,000, R&D Systems, AF1979), pH3 (rabbit, 1:500, Millipore, 06-570), PITX3 (goat, 1:500, SantaCruz, sc-19307), PDGFRa (rabbit, 1:100, Cell Signaling, 5241), SOX2 (rabbit, 1:500, Millipore, AB5603), TH (rabbit, 1:1,000, Millipore, AB152), TH (mouse, 1:500, ImmunoStar, 22941), and TH (sheep, 1:500, Novus, NB300).

EdU pulse and chase

Click-iT EdU Imaging Kit (Thermo Fisher Scientific) was used for EdU pulse and chase experiment.10 μM EdU was supplemented into the culture medium for 4 hr at day 16 of differentiation, and then the cells were cultured until day 21. After fixation by 4% PFA, EdU detected was performed according to the manufacturer's protocol.

Single-cell RNA-sequencing libraries preparation

hESCs and cells of differentiation days 11, 16, 21, 28 were thawed and re-suspended in NeutriStem medium with 10 μM Y27632. Cells were centrifuged at 300 x g for 2 min. Cell pellets were re-suspended in BD staining buffer (FBS) at room temperature. Cell suspension was mixed with Sample Tag and incubated at room temperature for 20 min. Cells were washed with BD staining buffer twice and re-suspended in PBS (with 0.04% BSA). Cells were filtered and counted. For each hESC line, we pooled 1000 cells from day 0, 1200 cells from differentiation days 11, 16 and 21, and 1400 cells from day 28. The pooled cells were processed with single cell capture, reverse transcription, and cDNA amplification according to the 10x Chromium™ Single Cell 3' Reagent Kits v2 User Guide. The corresponding Sample

Tag libraries were prepared according to BD Single-Cell Multiplexing Kit—Human.

Processing of single cell data

The samples were aligned to a combined reference genome of GRCh38.p12 and BD sample tags using Cell Ranger v3.0.2. Samples were then de-multiplexed using BD Genomics Sample Multiplexing tools v0.4 from Cell Ranger outputs. Final UMI count matrices were obtained by running Velocyto v0.17 with default parameters for Chromium 10X samples on de-multiplexed bam files with a gtf file combined of the human reference and BD sample tags. Data was filtered and processed using Cytograph (La Manno et al., 2016) with the following quality parameters. For each cell line count matrices, cells with less than 2000 UMIs detected transcripts, more than 5% of mitochondrial genes in their library, and identified as doublets by the Cytograph version of DoubletFinder, were excluded from further analysis. After filtering, there were 1359, 2372, 2714, 1962, 3274 cells remained at day 0, day 11, day 16, day 21 and day 28 of differentiation respectively with mean UMIs between 6 753 and 15 482, and mean detected transcripts between 2 490 and 3 931 (**Figure 5A**). Dimension reduction and Louvain clustering were then performed using Cytograph with PCA using 40 components on highly variable genes detected by variances. Clusters were refined by aggregating clusters that were not transcriptionally differentiated by manual inspection of gene enrichment for each cluster, resulting in 29 clusters (**Figure 5B**). Then, cells from days 21 and 28 of differentiation (cluster 11 onwards) were pulled for another iteration of processing as described. Final cluster membership (**Figure 5A**) was obtained by aggregating clustering results from the iterations.

Comparison of hESC-derived cells to the human ventral midbrain development reference dataset

UMI matrices of hESCs-derived cells from the current protocol were log-transformed, normalized and scaled to the reference data with selected gene set (described below). The same transformation was performed to UMI matrices from previous hESC-derived midbrain cells (La Manno et al., 2016), which were differentiated as described (Kriks et al., 2011). The similarities of *in vitro* cells to *in vivo* reference were measured as probabilities being each reference cell types, using logistic regression (described below) and visualized in a wheel plot as described (La Manno et al., 2016). In brief, the similarities to reference cell types are summarized as dot products to respective coordinates of reference cell types in the wheel, such that the distance to each reference cell type of an individual cell is in proportion to its relative similarity to the reference. For comparing the hESC-derived cells to the *in vivo* reference, L2-regularised logistic regression was used on human ventral midbrain cell types (La Manno et al., 2016), as well as mouse pericyte lineage cells (PLCs) and vascular leptomeningeal cells (VLMCs) (Marques et al., 2018). Logistic regression was implemented as described (La Manno et al., 2016), using the following prototypes: Embryonic stem cells (eES) consisting of eSCa, eSCb and eSCc; Floor plate progenitors (ProgFP) consisting of ProgFPM and ProgFPL; Radial glia 2 (Rgl2) consisting of hRgl2a, hRgl2b, hRgl2c; Dopaminergic

neurons (DA) consisting of hDA0, hDA1 and hDA2; GABAergic lineage (Gaba) consisting of hGaba and hNbGaba; VLMCs consisting of pnVLMCs and VLMCs; Pericytes (Peric) consisted of hPeric and PLCs.

Logistic regression training

For training the logistic regression, UMI matrices corresponding to cells belonging to cell types of interests (La Manno et al., 2016; Marques et al., 2018) were aggregated, after conversion of mouse genes to their human counterparts with Ensembl BioMart tool (GRCh37 version). Then a gene set (excluding sex, mitochondrial, erythrocytes and cell-cycle related genes) was selected from recursive feature elimination (RFE) (Guyon et al., 2002) using linear supper vector classification (SVC) with 5-fold stratified cross validation, step = 0.1, and F1-weighted score as the evaluation on log-transformed, max-normalized and median total UMIs-scaled data. The implementation was performed using the python package scikit-learn v0.23.1. With RFE, we have converged to a compact set of genes (n = 1184) that was discriminant between reference cell types, by eliminating incrementally 10% of the genes that were least important. To attest the performance of the selected gene set on cell type prediction, 80% of the normalized and scaled data was used for optimizing the regularization strength (C) with either the selected gene set or size-matched random gene set (n = 1184). For each condition, C was scanned using Optuna (Akiba et al., 2019), a Bayesian hyperparameter optimization, with the default sampler over log uniform distribution (between the range of 0.001 and 2) for 100 trials. Over the trials, C was optimized by evaluating the mean brier score of 5-folds train-test split per trial. Mean accuracy per trial was also logged (**Figure S5F**). The selected gene set was validated by using the optimized C to predict the 20% of the data at the end of the optimization (**Figure S5G** and **S5H**). Final model with the full dataset was trained in the same approach (C = 1.99) and used for comparing *in vitro* cells.

Analysis of dopamine release and content by HPLC

For dopamine measurement experiments, hESC-derived mDA progenitors were plated on LN511 coated 12-well plates at a density of 5 x 10^5 cells/cm^2 on day 16 and collected on days 28, 35, 42 and 56 of differentiation for dopamine contents and release. Cells were incubated in 200 µL of Neurobasal + N2 medium for 30 min at 37°C and the supernatant was collected. Cells were then incubated in Neurobasal + N2 medium supplemented with 56 mM KCl for 30 min at 37°C and the supernatant collected. The supernatant was immediately stabilized with 20 µL of 1 M perchloric acid containing 0.05% sodium metabisulphite and 0.01% ethylene-diamine-tetra-acetic acid (EDTA) disodium salt and the samples stored at -80°C. Two days later, cells were collected to measure intracellular dopamine content. On the day of analysis, samples were centrifuged at 16,000 x g for 10 min at 4°C and then filtered through 0.2 µm nylon membrane inserts by centrifugation at 4,000 x g for 5 min at 4°C. The HPLC-ECD system used was a Dionex Ultimate 3000 series (Dionex, ThermoFisher Scientific, USA) and the injection volume was 20 µL for each sample. Analyte separation was performed on a

Dionex C18 reversed-phase MD-150 3.2 mm x 250 mm column (3 µm particle size). Column and analytical cell were kept at 30°C and the first and second analytical cell were set to -100 mV and +300 mV, respectively. The mobile phase was pumped at a flow rate of 0.4 mL/min and consisted of 75 mM monobasic sodium phosphate, 2.2 mM 1-octanesulfonic acid sodium salt, 100 µL/L triethylamine, 25 µM EDTA disodium salt and 10% acetonitrile (v/v), pH 3.0 adjusted with 85% phosphoric acid. Chromatograms were acquired with Chromeleon software (Dionex, ThermoFisher Scientific) over an acquisition time of 55 min. Dopamine concentration was calculated for each sample. For dopamine release experiments, dopamine levels after KCl stimulation were normalized to un-stimulated dopamine levels. For intracellular dopamine content, dopamine content was normalized to intracellular dopamine content on day 28. Data is shown as averaged normalized values from three independent experiments.

Electrophysiological recordings

For patch-clamp electrophysiological recordings, hESC-derived mDA progenitors were plated on LN511 coated 24-well plates at a density of 500 000 cells/cm^2 on day 16 and recordings were performed between days 56-73 of the differentiation protocol. Cells with neurites and non-flat cell body, general morphological aspects of neurons, were selected for whole-cell patch clamp recordings. Borosilicate glass pipettes (4-10 MΩ) were filled with intracellular solution containing 105 mM K-gluconate, 30 mM KCl, 10 mM Na-phosphocreatine, 10 mM HEPES, 4 mM Mg-ATP, 0.3 mM Na-GTP, and 0.3 mg/mL of Lucifer yellow (Sigma-Aldrich) (pH 7.3 adjusted with KOH). The cells were continuously perfused with a solution containing 140 mM NaCl, 2.5 mM KCl, 1.2 mM NaH_2PO_4, 1 mM $MgSO_4$, 1.3 mM $CaCl_2$, 10 mM glucose and 10 mM HEPES, pH 7.4, bath kept 30-35˚C. The signal was amplified and digitized with Multiclamp 700B (Molecular Devices) and Digidata 1550 (Molecular Devices), respectively. Clampfit 11.1 (Molecular Devices) was used for analysis. For sEPSC detection, cells were clamped to -70 mV in voltage-clamp mode and 10-30 seconds were analyzed per cell by using template search (template made by averaging 10 typical EPSCs), false positives were manually removed by an analyzer blinded for the DIV of the cells. The remaining recordings were in current-clamp mode. Input resistances were calculated with a 1 second long ± 4-20 pA current injection. Spontaneous AP frequency was assessed during 30-60 seconds gap free recording. For spiking properties, cells that had a resting membrane potential above spiking threshold were brought down to ca -70 mV before current step injections. Pearson's correlation (r) and two-tail p-values were reported.

Statistic analysis

Results are given as means ± standard deviation (SD) or standard error of the mean (SEM). The significance of differences was determined by Student's *t*-test for single comparisons and by one-way analysis of variance (ANOVA) or two-way ANOVA for multiple comparisons. Further statistical analysis for *post hoc* comparisons was performed using Tukey's test (Prism 6; GraphPad, San Diego, CA, USA). Mann-Whitney U-test was also performed using R v4.1.1

to analyze the numbers of VLMCs. Permutation test was performed using python v3.7.7 for analyzing the average gene expression for top-similar cells as shown from logistic regression analysis between current protocol and the protocol by Kriks et al., 2011.

Supplemental references

Akiba, T., Sano, S., Yanase, T., Ohta, T., and Koyama, M. (2019). Optuna: A next-generation hyperparameter optimization framework. Proc. ACM SIGKDD Int. Conf. Knowl. Discov. Data Min. 2623–2631.

Guyon, I., Weston, J., Barnhill, S., and Vapnik, V. (2002). Gene selection for cancer classification using support vector machines. In Machine Learning, pp. 389–422.

Supplementary Table S1

List of primer sequences

**Gene**	**Forward**	**Reverse**
ABCA1	ATGTGAGGCGGGAAAGACAGAG	ATCCTGTCAACAGCAGGCTTCC
ALDH1A1	TGTTAGCTGATGCCGACTTG	CTGGCCCTGGTGGTAGAATA
BARHL1	CCAGAACCGCAGGACTAAATGG	CTGGAGCGCTGAGTAATTGCCT
CALB1	GACGGAAGTGGTTACCTGGA	TGCCCATACTGATCCACAAA
CORIN	CATATCTCCATCGCCTCAGTTG	GGCAGGAGTCCATGACTGT
DCX	ACCTCCAGCAGCCAGCTCTCTA	GGCAGGTACAGGTCCTTGTGCT
DEAF1	*CCAGGTCCTCAGTCTCCTCCAA*	TGTCGTACACAGAAGGGTCCCA
DKK3	*GGTGGAAGAGATGGAGGCAGAA*	*CCAACCTTCGTGTCTGTGTTGG*
EBF1	GTGCGAGTTCATCGTCTGAGA	ACTTGTATCAGATTACTCTC
EBF2	GATTTGCTGGCAACGTTGGG	TCATTATTGGTCCATCAGAG
EN1	CGTGGCTTACTCCCCATTTA	TCTCGCTGTCTCTCCCTCTC
ERBB4	TGGCCACCAAACATGACTGACT	GAGAGGTGATGCCCTGTTGCTT
FGF8B	AGGTAACTGTTCAGTCCTCACC	TGTAGAGTTGGTAGGTCCGG
FOXA2	TTCAGGCCCGGCTAACTCT	AGTCTCGACCCCCACTTGCT
FOXG1	GCTGGACATGGGAGATAGGA	GTGGTGGTTGTCGTTCTGG
GAPDH	TTGAGGTCAATGAAGGGGTC	GAAGGTGAAGGTCGGAGTCA
GBX2	GTTCCCGCCGTCGCTGATGAT	GCCGGTGTAGACGAAATGGCCG
KCNJ6	TAGAGGACCCCTCCTGGACT	TCCCTCTGGGCATTTATCTG
HOXA2	AGTCTCGCCTTTAACCAGCA	TAGGCCAGCTCCACAGTTCT
LMO3	CTCTCAGTCCAGCCAGACACCA	GGCACACTTCAGGCAGTCTTCA
LMX1A	GATCCCTTCCGACAGGGTCTC	GGTTTCCCACTCTGGACTGC
MSX1	CGAGTTAAAGATGGGGAAACTG	GAGACATGGCCTCTAGCTCTGT
NANOG	ACAACTGGCCGAAGAATAGCA	GGTTCCCAGTCGGGTTCA
NHLH1	CCCGACAAGAAGCTCTCCAAGA	CAGGCTGAGTTCAGACGTCCAG
NGN2	GCTGGGTCTGGTACACGATT	GGCCTTCAGTCTACGGGTCT
NKX2.1	AGAGGGCTCTGTGCTGACAT	CAGAGTGTGCCCAGAGTGAA
NEUROD1	ACCCCTACTCCTACCAGTCGCC	GGCTTAACGTGGAAGACATGGG
NR4A2	CAGCTCCGATTTCTTAACTCCAG	GGTGAGGTCCATGCTAAACTTGA
OTX2	ACAAGTGGCCAATTCACTCC	GAGGTGGACAAGGGATCTGA
PBX1	TAAAAAGCCTTGGTGCTTCCCA	GCTCGTCCATCTCCAAAGGCTA
PITX2	CATGTCCACACGCGAAGAAATC	CCCGACGATTCTTGAACCAAAC
POU5F1	AGGGCCCCATTTTGGTACC	TCAGTTTGAATGCATGGGAGAGC
POU6F1	GCCTACAGCCAGTCAGCCATCT	GTTCCGCAGTTCAGCTTCGTTT
SIX3	AACTTCCGCGACCTCTACCACA	AGCTTCTCGGCCTCCTGGTAGT
SOX2	CAAGATGCACAACTCGGAGA	GCTTAGCCTCGTCGATGAAC
SREBF1	AACACAGACGTGCTCATGGAGG	CTCTGGAAAGGTGAGCCAGCAT
TH	ACTGGTTCACGGTGGAGTTC	TCTCAGGCTCCTCAGACAGG
TUBB3	CATTCTGGTGGACCTGGAAC	ATACTCCTCACGCACCTTGC
WNT1	GAGCCACGAGTTTGGATGTT	TGCAGGGAGAAAGGAGAGAA
WNT5A	ACTGCAAGTTCCACTGGTGCTG	GTGGCACCCACTACTTGCACAC
WNT7A	CAATCGGGACTATGAACCGGAA	GCCCAGAGCTACCACTGAGGAG
WNT11	GAAGCGACAGCTGCGACCTTAT	CAGGTGACGTAGCAGCACCAGT
